# Supplementary material for: Emotional distress and well-being among people with motor neurone disease (MND) and their family caregivers: a qualitative interview study
Source: BMJ Open. 2021 Aug 16;11(8):e044724. doi: 10.1136/bmjopen-2020-044724 (PMC8372816; doi:10.1136/bmjopen-2020-044724)
Supplement: Supplementary data [file bmjopen-2020-044724supp001.pdf]

SUPPLEMENTARY FILE 1

2. Consolidated criteria for reporting qualitative studies (COREQ):

Tong A, Sainsbury P, Craig J. Consolidated criteria for reporting qualitative research (COREQ): a 32-item checklist for interviews and focus groups. *International Journal for Quality in Health Care*. 2007. Volume 19, Number 6: pp. 349 – 357

No. Item	Guide questions/description	Reported on Page #
**Domain 1: Research team and reflexivity**		
Personal Characteristics		
1. Interviewer/facilitator	Which author/s conducted the interview or focus group?	CP – Methods section, Pg 7
2. Credentials	What were the researcher's credentials? E.g. PhD, MD	PhD student – Methods section, Pg 8
3. Occupation	What was their occupation at the time of the study?	PhD student – Methods section, Pg 8
4. Gender	Was the researcher male or female?	Female
5. Experience and training	What experience or training did the researcher have?	Methods section – Pg 7-8
Relationship with participants		
6. Relationship established	Was a relationship established prior to study commencement?	No, Pg 6
7. Participant knowledge of the interviewer	What did the participants know about the researcher? e.g. personal goals, reasons for doing the research	Participants knew that the researcher was a PhD student at the University of Southampton, doing her PhD on emotional distress and wellbeing in Motor Neurone Disease (MND). This information was described on the participant information sheets and

		study adverts. Pg 6
8. Interviewer characteristics	What characteristics were reported about the interviewer/facilitator? e.g. Bias, assumptions, reasons and interests in the research topic	The interviewer did not have much prior knowledge of MND, but had previously worked with and interviewed people with Parkinson's disease and people receiving palliative care. Pg 8
**Domain 2: study design**		
Theoretical framework		
9. Methodological orientation and Theory	What methodological orientation was stated to underpin the study? e.g. grounded theory, discourse analysis, ethnography, phenomenology, content analysis	The study used methods (in-depth semi-structured interviews and reflexive thematic analysis) in line with an interpritivist paradigm. Pg 6
Participant selection		
10. Sampling	How were participants selected? e.g. purposive, convenience, consecutive, snowball	Purposive sampling – Methods section, Pg 6
11. Method of approach	How were participants approached? e.g. face-to-face, telephone, mail, email	Methods section – Pg 6-7
12. Sample size	How many participants were in the study?	Methods section – Pg 9
13. Non-participation	How many people refused to participate or dropped out? Reasons?	Participants contacted the researcher if they wanted to take part in an interview. No one dropped out. Pg 9
Setting		
14. Setting of data collection	Where was the data collected? e.g. home, clinic, workplace	Methods section – Pg 7
15. Presence of non-participants	Was anyone else present besides the participants and researchers?	No (not reported because not present)
16. Description of sample	What are the important characteristics of the sample? e.g. demographic data, date	Methods section – Pgs 9-10
Data collection		

17. Interview guide	Were questions, prompts, guides provided by the authors? Was it pilot tested?	Methods section, Pg 7 and supplementary file 2
18. Repeat interviews	Were repeat interviews carried out? If yes, how many?	N/A
19. Audio/visual recording	Did the research use audio or visual recording to collect the data?	Methods section – Pg 7
20. Field notes	Were field notes made during and/or after the interview or focus group?	Field notes were made after each interview. Pg 7
21. Duration	What was the duration of the interviews or focus group?	Average duration reported – Methods section, Pg 9
22. Data saturation	Was data saturation discussed?	No. We were not aiming for theoretical data saturation, but rather to look at patterns of experience across participants with diverse characteristics. (not reported because not applicable to this study)
23. Transcripts returned	Were transcripts returned to participants for comment and/or correction?	No (not reported because was not carried out)
**Domain 3: analysis and findings**		
Data analysis		
24. Number of data coders	How many data coders coded the data?	One – Methods section, Pgs 7-8
25. Description of the coding tree	Did authors provide a description of the coding tree?	N/A
26. Derivation of themes	Were themes identified in advance or derived from the data?	Inductive thematic analysis – Methods section, Pg 7
27. Software	What software, if applicable, was used to manage the data?	NVivo Pg 8
28. Participant checking	Did participants provide feedback on the findings?	Not participant checking, but lay summary of findings was sent to all participants Pg 8
Reporting		

29. Quotations presented	Were participant quotations presented to illustrate the themes/findings? Was each quotation identified? e.g. participant number	Yes, Results section Pgs 9-21
30. Data and findings consistent	Was there consistency between the data presented and the findings?	Yes, illustrative quotations have been provided for each theme and sub-theme – Results section Pgs 9-21
31. Clarity of major themes	Were major themes clearly presented in the findings?	Results section and presented in Figures 1 & 2, Pgs 9-21
32. Clarity of minor themes	Is there a description of diverse cases or discussion of minor themes?	Description of diverse cases embedded within the explanation of themes – Results section Pgs 9-21
