# Supplementary material for: Emotional distress and well-being among people with motor neurone disease (MND) and their family caregivers: a qualitative interview study
Source: BMJ Open. 2021 Aug 16;11(8):e044724. doi: 10.1136/bmjopen-2020-044724 (PMC8372816; doi:10.1136/bmjopen-2020-044724)
Supplement: Supplementary data [file bmjopen-2020-044724supp002.pdf]

SUPPLEMENTARY FILE 2

1. Interview topic guide

(1) Can you tell me about your experience with MND?

- wherever the participant would like to start, could be about diagnosis or their experience now.
- Have your thoughts and feelings changed since you were diagnosed or are they the same? (If different, in what way have they changed?)

(2) What's it like to live with MND? Can you tell me a bit more about that?

- What are your main concerns/worries/emotions you face on a day to day basis?
- What are your thoughts and feelings about living with MND?

(3) Can you tell me about how you manage MND on a daily basis? If you find yourself having emotional concerns or worries, do you have any ways of managing them?

(4) Can you tell me about any support you feel you have? Or people/ things/ processes that have helped you?

(5) (In addition to this support) Do you have any thoughts about what else might support you to cope with MND?

- particularly additional support with any emotional concerns/worries that participants have previously mentioned.
