# Supplementary material for: Emotional distress and well-being among people with motor neurone disease (MND) and their family caregivers: a qualitative interview study
Source: BMJ Open. 2021 Aug 16;11(8):e044724. doi: 10.1136/bmjopen-2020-044724 (PMC8372816; doi:10.1136/bmjopen-2020-044724)
Supplement: Author's manuscript [file bmjopen-2020-044724.draft_revisions.pdf]

BMJ Open is committed to open peer review. As part of this commitment we make the peer review history of every article we publish publicly available.

When an article is published we post the peer reviewers' comments and the authors' responses online. We also post the versions of the paper that were used during peer review. These are the versions that the peer review comments apply to.

The versions of the paper that follow are the versions that were submitted during the peer review process. They are not the versions of record or the final published versions. They should not be cited or distributed as the published version of this manuscript.

BMJ Open is an open access journal and the full, final, typeset and author-corrected version of record of the manuscript is available on our site with no access controls, subscription charges or pay-per-view fees (http://bmjopen.bmj.com).

If you have any questions on BMJ Open's open peer review process please email info.bmjopen@bmj.com

BMJ Open

Emotional distress and wellbeing among people with Motor Neurone Disease (MND) and their family caregivers: a qualitative study

Journal:	*BMJ Open*
Manuscript ID	bmjopen-2020-044724
Article Type:	Original research
Date Submitted by the Author:	12-Sep-2020
Complete List of Authors:	Pinto, Cathryn; University of Southampton, Psychology<br>Geraghty, Adam; University of Southampton, Primary Care and Population Sciences<br>Yardley, Lucy; University of Southampton, Psychology; University of Bristol, School of Health Sciences<br>Dennison, Laura; University of Southampton
Keywords:	Motor neurone disease < NEUROLOGY, Depression & mood disorders < PSYCHIATRY, Anxiety disorders < PSYCHIATRY, QUALITATIVE RESEARCH

SCHOLARONE™
Manuscripts

I, the Submitting Author has the right to grant and does grant on behalf of all authors of the Work (as defined in the below author licence), an exclusive licence and/or a non-exclusive licence for contributions from authors who are: i) UK Crown employees; ii) where BMJ has agreed a CC-BY licence shall apply, and/or iii) in accordance with the terms applicable for US Federal Government officers or employees acting as part of their official duties; on a worldwide, perpetual, irrevocable, royalty-free basis to BMJ Publishing Group Ltd ("BMJ") its licensees and where the relevant Journal is co-owned by BMJ to the co-owners of the Journal, to publish the Work in this journal and any other BMJ products and to exploit all rights, as set out in our licence.

The Submitting Author accepts and understands that any supply made under these terms is made by BMJ to the Submitting Author unless you are acting as an employee on behalf of your employer or a postgraduate student of an affiliated institution which is paying any applicable article publishing charge ("APC") for Open Access articles. Where the Submitting Author wishes to make the Work available on an Open Access basis (and intends to pay the relevant APC), the terms of reuse of such Open Access shall be governed by a Creative Commons licence – details of these licences and which Creative Commons licence will apply to this Work are set out in our licence referred to above.

Other than as permitted in any relevant BMJ Author's Self Archiving Policies, I confirm this Work has not been accepted for publication elsewhere, is not being considered for publication elsewhere and does not duplicate material already published. I confirm all authors consent to publication of this Work and authorise the granting of this licence.

For peer review only

Emotional distress and wellbeing among people with Motor Neurone Disease (MND) and their family caregivers: a qualitative study

Cathryn Pinto,[a]* Adam W A Geraghty[b], Lucy Yardley[a,c] and Laura Dennison[a]

[a]*Department of psychology, University of Southampton, Southampton, UK;*

[b]*Primary care, population sciences and medical education, University of Southampton, Southampton, UK;*

[c]*School of psychological science, University of Bristol, Bristol, UK*

*Corresponding author: Cathryn Pinto, University of Southampton, B44 Highfield campus, Southampton SO17 1BJ Email: C.L.Pinto@soton.ac.uk

Word count: 3,876

For peer review only

Abstract

Objective: We aimed to get an in-depth understanding of the emotions experienced by people with MND and their caregivers and to explore what impacts emotional distress and wellbeing.

Design: Qualitative study using semi-structured interviews with people with MND and caregivers.

Setting: Participants were recruited from across the UK and took part in interviews conducted either face-to-face, by telephone or email to accommodate for varying levels of disability.

Participants: 25 people with MND and 10 caregivers took part. Participants were purposively sampled based on their MND diagnosis, symptoms and time since diagnosis.

Data analysis: Data were analysed using inductive reflexive thematic analysis.

Results: Eight broad themes were generated (20 subthemes). Participants described the emotional distress of losing physical function and having a threatened future because of poor prognosis. Keeping up with constant changes in symptoms and feeling unsupported by the healthcare system added to emotional distress. Finding hope and positivity, exerting some control, being kinder to oneself, and experiencing support from others were helpful strategies for emotional wellbeing.

Conclusion: We provide an in-depth understanding of what impacts emotional distress and wellbeing and discuss implications for psychological interventions for people with MND and their family. Any communication and support provided for people with MND and their caregivers, needs to pay attention to concepts of hope, control, and compassion.

For peer review only

Keywords: Motor neuron disease; emotional distress; wellbeing; depression; qualitative research

Article summary (Strengths and Limitations):

- This study focuses on the experience of emotions in people with MND and their caregivers and provides an in-depth account of what impacts emotional distress and wellbeing.
- Purposive sampling and flexible recruitment strategies used to capture experiences of emotional distress and wellbeing from people with MND with various symptoms and at differing lengths of time since diagnosis.
- Findings did not represent the experiences of people with mild cognitive impairment, who were underrepresented in the sample.

For peer review only - http://bmjopen.bmj.com/site/about/guidelines.xhtml

INTRODUCTION

Motor neurone disease (MND) is a neurodegenerative disease, which results in declining physical function and has a very poor prognosis.(1) People with MND (pwMND) also experience a range of psychological symptoms. Many pwMND experience depression.(2-4) Anxiety is prevalent around the time of diagnosis(5, 6) and during the final stages.(7) PwMND also experience other forms of emotional distress besides anxiety and depression including feelings of hopelessness(8-10) and demoralization.(2) Emotions are also affected by low self-esteem,(4, 11) end of life concerns,(8) faith/existential concerns,(12, 13) sense of loss,(13, 14) and changes in identity, roles and relationships.(11, 13, 15) Qualitative research on experiences of living with MND has also described the impact of the significant losses that come with MND on personal, social and occupational relationships.(10) Family members or caregivers of pwMND often struggle with the emotional impact and burden of the disease, and have high rates of psychological morbidity.(16-18) Qualitative studies with caregivers have described the strain from caring tasks, from having to make changes to their own lives and not having time for themselves.(19-21) In terms of wellbeing, psychosocial factors like coping strategies and social support, are more strongly related to wellbeing and quality of life than demographic and clinical factors.(11, 22, 23)

There have however, been few effective psychological interventions to improve psychological wellbeing,(24) and more research and interventions are needed.(17) An in-depth understanding of the emotions experienced, factors that trigger emotional distress, ways in which people cope with emotional distress and improve wellbeing would help inform future psychological interventions.

Qualitative research with pwMND and their caregivers can provide such information. Few qualitative studies have specifically examined emotional distress and wellbeing in MND and

For peer review only - http://bmjopen.bmj.com/site/about/guidelines.xhtml

potential factors that may impact distress or wellbeing. One study examined the use of metaphors to express emotions,(25) and a small number have focused on emotions at specific time periods (e.g. during diagnosis or the final stages).(6, 14, 26) It is also important to understand the experiences of emotions and coping among people whose experiences are not yet well represented in research, especially people with speech difficulties, cognitive impairment, and people at different stages of the disease.

Our aim is to understand the emotional impact of living with MND and to explore what impacts experiences of emotional distress and wellbeing among pwMND and their family caregivers.

For peer review only

METHODS

Design

Qualitative study using in-depth semi-structured interviews, reported in accordance with the Consolidated criteria for reporting qualitative research (COREQ) (see Supplementary file 2).(27)

Participants

Participants were 25 pwMND and 10 family caregivers, purposively sampled to include people with varying types and severity of symptoms, and time since diagnosis (see Table 1). Participants were above 18 years of age, had an MND diagnosis, and had mental capacity to consider participation in the study. We included participants who self-reported difficulties with cognition, but had mental capacity to give informed consent, as the views of pwMND who have cognitive impairment have been underrepresented in previous research. We included caregivers above 18 years of age, both current and recently bereaved (bereavement within 1 year from the time of the interview).

Table 1: Demographic and clinical details of the sample

Characteristic	People with MND (n=25)	Caregivers (n=10)
**Age (range 39-80)**		
35-50 years	3	0
51-65 years	12	4
66-80 years	10	6
**Gender**		
Male	15	5
Female	10	5

For peer review only - http://bmjopen.bmj.com/site/about/guidelines.xhtml

**Relationship to person with MND**		
Spouse/partner		10
**Diagnosis**		
ALS limb	18	
ALS bulbar	1	
Primary Lateral Sclerosis	4	
Progressive Bulbar Palsy	1	
Progressive Muscular Atrophy	1	
**Time since diagnosis (range 2 months-17 years)**		
Less than 1 year	8	
1-3 years	6	
3-5 years	7	
More than 5 years	4	
**Difficulties reported often/always *(ALSAQ-5 scale)**		
Difficult to stand up	16	
Difficult to use arms and hands	16	
Difficulty eating solid food	4	
Speech is not easy to understand	9	
Feeling hopeless about the future	3	
**Self-reported concerns about cognitive ability**	2	

*Amyotrophic Lateral Sclerosis Assessment Questionnaire (ALSAQ-5) is a patient self-report questionnaire used to briefly measure the impact of ALS/MND on patients.(28)

For peer review only

Data collection

Participants were recruited through a UK charity that supports pwMND and their families (Motor Neurone Disease Association). Study information was circulated via the charity's website, newsletters, social media outlets and local support groups. People willing to take part contacted the lead author (CP), who screened for eligibility and provided further details about the interview.

29 interviews were conducted in total. Where two members of a couple had both consented to participate participants were interviewed separately where possible, but jointly where this was requested for reasons of comfort or to facilitate easier communication (n=6). Interview mode was also flexible; 14 interviews were conducted face-to-face, 8 via telephone, and 7 via email, to accommodate various symptoms and levels of disability. Before each interview, participants gave written informed consent and filled a demographic/clinical details form. Face-to-face interviews were conducted in a place convenient to participants, usually at home or a hospice. The interview topic guide (Supplementary file 1) covered questions about people's experiences living with MND, with a focus on their thoughts and feelings and coping with emotional concerns. Interviews lasted an average of 39.6 minutes.

Data analysis

Face-to-face and phone interviews were audio recorded and transcribed, and all interviews were anonymized. Data were analyzed using reflexive thematic analysis,(29, 30) as this was a flexible method that suited the research question, helped us identify common patterns across participants' experiences and allowed us to look for underlying meaning behind experiences of emotional distress and wellbeing. The analysis was mainly conducted by CP, a PhD

For peer review only - http://bmjopen.bmj.com/site/about/guidelines.xhtml

student with previous experience as a qualitative researcher. After familiarisation with the interview transcripts, Nvivo 12 software was used to code the data inductively, focusing on semantic and latent features of the data. The codes went through several iterations as new interviews were coded. During the coding process, notes were made about interesting features of the data and how different concepts related to each other. Following this, similar codes about emotional distress and emotional wellbeing were grouped together and candidate themes were generated. Themes were then reviewed to see if they represented experiences across the dataset. Theme names were revised and findings written up; this was also an iterative process whereby descriptions were clarified and overlap between themes identified. CP had regular meetings with qualitative research experts LD and AG who helped refine codes and themes.

Ethics

We obtained ethics from the University of Southampton ethics committee (ERGOII-46996). Written informed consent was obtained for all participants. Participants were debriefed and steps were taken following each interview to ensure participants were not distressed.

Patient and Public Involvement (PPI)

Three PPI members (one person with MND and 2 former caregivers) contributed to this study. They were involved in the recruitment stage to help the researcher identify potential participants from local groups. PPI members also contributed to piloting the interview topic guide, refining interview questions and trialling the data collection procedure to ensure that it was not burdensome. Some PPI members also looked over the early drafts of the findings and offered insight about elements to highlight or discuss in the reporting of the results.

For peer review only - http://bmjopen.bmj.com/site/about/guidelines.xhtml

For peer review only

RESULTS

We developed 8 themes and 20 subthemes. The first four themes relate to triggers of emotional distress (Fig 1); the remaining four themes capture strategies to improve emotional wellbeing (Fig 2). All themes were present in some degree and form in both patient and caregiver interviews; where differences between these groups were apparent, they are described.

[Insert Fig 1 here]

Triggers of emotional distress

Participants described 4 main triggers of emotional distress and how they had an impact on emotions (themes are numbered, *subthemes in italics*).

(1) Losing function or ability

Most participants described how losing function or ability was distressing because of a *reduced sense of autonomy and control.* PwMND spoke about feelings of anger, frustration, sadness or grief at not being able to do the things they wanted to, at losing their independence and relying on others for help. Similarly, caregivers expressed sadness at seeing their family member lose capability and independence.

> "Hobbies that I had, I can't pursue any of them because I just don't have any capability enough in my arms now to do that. So, I think mentally I've seen a dip in the last six months into a much more kind of negative and angry stage really." (Person with MND, P13)

For peer review only

> "...and seeing him, and it saddened me, where you're sort of doing the job of hoisting and moving, the manual handling, and then you step back and you're seeing it from a distance, that's actually quite [pauses] hard as well, because he's got no control over his body and he's having to have people do that for him." (Caregiver, C05)

Losing function led to *changes in identity*, specifically those of becoming a 'patient' and 'carer'. These changes, especially losing one's voice which is strongly linked to identity, were experienced as distressing.

> "Losing mobility gradually was bad enough… but losing my voice is much more dreadful. It's taking away part of my personality. I can no longer express myself properly." (Person with MND, P21)

Losing function or ability sometimes led to *changes in relationships and interactions*. PwMND spoke about negative feelings of becoming a burden for their partner/spouse. Both patient and caregiver participants spoke about how having MND sometimes changed or limited interactions between family members.

> "It's emotionally difficult not being able to physically help my son or my parents and siblings and friends. Not being able to hold and play with my nephew." (Person with MND, P26)

Participants also had *practical concerns or worries about managing tasks* as a result of losing more ability and function.

For peer review only - http://bmjopen.bmj.com/site/about/guidelines.xhtml

> "My main worry at the moment really is moving him from there to there because his knees and lower legs are getting weaker and I've got that [points to hoist] to move him." (Caregiver, C03)

(2) Having a threatened future

The short prognosis that accompanies a diagnosis of MND led to feelings of anger, disappointment, sadness and feelings of being robbed/cheated of a future (*threat to life narrative and future plans*).

> "But I'm having to accept I might have a lonely old age. Disappointment, more than anything else.' (Caregiver, C27)

Participants also described emotional distress when *anticipating future symptoms or disease progression*. Many things triggered worry about the future including researching MND online, meeting other pwMND, discussions with healthcare professionals and going through symptom checklists. Some participants also spoke about experiencing such worries about the future even if symptoms had not progressed or were relatively stable. This was true particularly just after diagnosis, where worries about the future led to feelings of depression, low mood and withdrawal from others.

> "Even though physically, say that first month afterwards, there was almost no change but my mind's thinking quite morose almost. You almost think of death at that point... It's not even anger at that stage, it's just hopelessness at that point because you're just thinking oh that is it isn't it? It's all over." (Person with MND, P10)

For peer review only - http://bmjopen.bmj.com/site/about/guidelines.xhtml

The *uncertainty of the disease progression* also caused emotional distress among both patients and caregivers, because of being unable to manage symptoms or plan for the future.

> "I think the biggest thing about this is that it's such an unknown. Because everybody is different and, who knows how much, how long this disease is going to affect you. And the uncertainty, for me, it's very hard to cope with, because you can't make plans.' (Caregiver, C15)

(3) Keeping up with constant and multiple changes

Participants discussed how the *timing and relentless nature of changes* in symptoms was difficult physically and emotionally. As time went on, for some it became easier to fall into a care routine. However, if deterioration happened quickly, both patient and caregivers expressed that it was difficult to cope and left them feeling tired, not in control and in need of respite.

> "If something's difficult, we find a way round it. And by the time we've found a way round it, things have moved on again, and it doesn't work. So that's very frustrating. And a bit… not depressing… soul destroying, because we're always playing catch up." (Person with MND, P24)

Sometimes physical symptoms necessitated *changes in many areas of life.* This included changes to their home, work, social and leisure activities. Both pwMND and caregivers spoke about the effort involved in either living with MND or looking after someone with MND. This sometimes affected thoughts and mood negatively including feelings of frustration,

For peer review only - http://bmjopen.bmj.com/site/about/guidelines.xhtml

anxiety, tiredness or strain/burden. For example, C03, a caregiver, recalled being upset and needing someone to talk to due to exhaustion from physically moving her spouse; "I used to get so tired that sometimes when he had gone to bed in the evening I did just feel like standing there crying."

(4) Feeling unsupported by professionals or the care system

Participants reported feeling distressed when healthcare professionals or the care system did not support their needs. *Infrequent or inefficient care provision* left people feeling abandoned and left on their own to deal with MND.

> "I felt completely unsupported by the neurologist or the health service, we were really just left on our own, completely in the dark as to what was gonna happen... So, it was a very, very distressing time." (Person with MND, P14)

Poor coordination between different professionals/organizations, difficulty accessing equipment and financial benefits also triggered feelings of frustration, injustice and being 'let down' by the system.

Poor or insensitive communication from healthcare professionals added to emotional distress. In particular, participants described upset as a consequence of a lack of empathy from professionals, or insensitive or unsupportive presentation of care.

> "Our life was shattered in 2 minutes. And I felt that was cruel, very very cruel [pause]. There should be a way of discussing it with you and saying "well look, you have such and such a condition, but hey, this is what we can do."

For peer review only - http://bmjopen.bmj.com/site/about/guidelines.xhtml

> Not just you've got this and throw you out, and so we had to find our own way round this problem." (Caregiver, C01)

[Insert Fig. 2 here]

Strategies used to improve emotional wellbeing

Participants described strategies they used to improve emotional wellbeing (themes are numbered; *subthemes in italics*).

(1) Finding hope and positivity

Despite the many losses, participants actively found ways to introduce hope and positivity. For many people, acceptance of MND was a gradual, learned process and even once there was acceptance, an attitude of not giving up on life was important for coping (*acceptance and not giving up)*. For some, not giving up included hoping for a cure or trying alternative therapies. For others, not giving up included finding solutions to make life easier, and not letting MND define them as they continued normal and valued activities. This strategy was endorsed mainly by pwMND.

> "I try to not challenge myself, but make sure there's nothing I'm not doing because of it. Almost in a way to say, it's not going to control me or control what I do." (Person with MND, P18)

For peer review only - http://bmjopen.bmj.com/site/about/guidelines.xhtml

Despite severe losses of function, many participants continued *doing activities that they enjoyed and that were meaningful*. Planning ahead and having things to look forward to in the immediate future added to feelings of hope.

> "Key thing is to have lots of little goals, lots of little hopes of things to do, normalities, weekends away, holidays but not in the far future. Everything's in a couple of weeks, 2-3 weeks, something to look forward to… it keeps the mind occupied to know that there is something coming up." (Person with MND, P10)

Most participants tried to *focus on the positive aspects of life*. This strategy became difficult in the context of increasing loss, but participants still stressed that it was important.

> "I'm still sad that I can't do the things that I used to be able to do. But you do learn to try and be positive about living with the condition as best you can and to live your life as well as you can." (Person with MND, P14)

To stay positive and deal with negative emotions, participants tried to reframe difficult situations by focusing on what they still can do, using humour, feeling grateful for symptoms they did not have or feeling lucky because of their circumstances or because of the support they received.

(2) Exerting some control

When MND took some control away, participants found other ways to exert control over their lives. Although a sense of control was important for most participants, they exerted this

For peer review only - http://bmjopen.bmj.com/site/about/guidelines.xhtml

control in very different ways. One way was by *focusing on the present moment* and making the most of what they could currently do. This strategy was adopted mainly by pwMND; it stopped them from worrying about the future or feeling sad for what they could no longer do, and instead had a more positive impact on emotions.

> "I realised there's absolutely no point in dwelling on the past because there's absolutely nothing I can do to get that back. So, it's waste of energy and there's no point in worrying about the future because there's absolutely nothing I can do about it. So, what I kind of taught myself to do is try to live in here and now because I do have some control over that." (Person with MND, P04)

Some participants described how it was helpful to exert *control over decisions about their care,* organizing or planning care and taking a more active problem-solving approach. This gave people a sense of empowerment when they would otherwise be struggling to cope, and also helped people hold onto their independence or identity despite the loss.

> "I hate the fact that I can't talk. Although I can talk, but it's a bit drained and doesn't sound like me. But I've done voice banking and I think you've got to be a step ahead." (Person with MND, P19)

However, some people exerted control by avoiding interactions or information that might remind them of future symptoms or deterioration and associated negative emotions.

For peer review only - http://bmjopen.bmj.com/site/about/guidelines.xhtml

"Everyone's different, aren't they? And their MND is different. Some people like to know the ins and out, but I don't know, I like to bury my head in the sand. Because I know that I would sit and worry about it all the time. And I don't want to." (Person with MND, P24)

(3) Being kinder to oneself

This strategy was mentioned by both pwMND and caregivers but for different kinds of tasks. Living with MND was often described as overwhelming and participants expressed the need for *taking a break* from MND. Caregivers spoke about the need to take a physical break from MND care; while pwMND spoke about taking a break from MND by doing regular activities and not thinking about MND.

"I'll have days where I deliberately avoid looking at anything and trying just to have days or a couple of days where I don't think about MND." (Person with MND, P18)

Some participants also expressed the need to give themselves *time and space to adjust to changes* in symptoms. This was done by allowing themselves space to vent if they felt overwhelmed, pacing their activities, lowering expectations, modifying how tasks were done, or asking for help.

"Keep doing the things you enjoy, just lower your expectations and standards so you achieve and don't feel disappointed." (Person with MND, P21)

(4) Experiencing support from others

For peer review only - http://bmjopen.bmj.com/site/about/guidelines.xhtml

Emotional and practical support from healthcare professionals, friends and family helped pwMND and caregivers cope with any emotional distress. Emotional support included being empathic, listening, being encouraging and positive. Participants also valued support from other people with MND because they felt truly understood and less alone.

> "I had a confirmation diagnosis at [hospital] and then I had a phone call from the [hospice] asking if I wanted to attend a kind of MND first contact group... and it's been a fantastic thing and I still go now. And that was really good, really supportive." (Person with MND, P19)

There were some differing views about the timing of offering support, especially just after diagnosis. Some participants wanted access to information and support almost immediately, however others needed some time to adjust before they accessed support.

Supportive communication from healthcare professionals and reliability of care helped patients and families feel reassured and confident about their care, which led to positive experiences and emotions. This included providing information based on the patient and families' readiness, communicating information in a sensitive and empathic manner, focusing on what can be done in terms of care and timely provision of care and equipment.

> "I would also mention my occupational therapist who has been brilliant at assessing my needs and getting in equipment quickly, usually just before they were needed. This has given me more confidence in the care and support I am given." (Person with MND, P22)

For peer review only - http://bmjopen.bmj.com/site/about/guidelines.xhtml

DISCUSSION

This study provides an in-depth understanding of the experience of emotional distress and emotional wellbeing among people with MND and their caregivers, and identifies triggers of distress and strategies used to improve wellbeing. Our findings come from a diverse sample, thereby highlighting key triggers of distress and coping strategies used by people with different abilities, symptoms and at different stages of having MND. PwMND and caregivers use coping strategies differently but still rely on similar concepts (hope, positivity, control, self-kindness, social support) to improve emotional wellbeing.

Some of our findings are in line with previous MND research, such as the distress caused by loss of ability, threats to the future,(10, 14, 15, 31) and not receiving appropriate professional support.(6, 7, 26, 32) One new finding in MND literature is the emotional distress triggered by multiple and constant changes brought about by disease progression and constantly 'playing catch up.' This has been described in research around caregiver experience;(33-35) we highlight how this is also distressing for pwMND. Interventions must consider this context of being overwhelmed by relentless change and ensure interventions are perceived as manageable, not burdensome.

We know that coping strategies are important for maintaining emotional wellbeing in MND,(23, 36, 37) and that hope and control are important for coping with MND.(10, 31, 38-40) Our findings go a step further to illustrate how these coping strategies relate to emotional wellbeing, particularly in the context of MND. For example, we illustrate the complexity of 'not giving up' and finding positivity in a degenerative disease where hope is constantly threatened. Our study also highlights the use meaning-based coping strategies (finding hope and positivity in the face of difficult circumstances and reappraising them in a positive way),

For peer review only - http://bmjopen.bmj.com/site/about/guidelines.xhtml

which generates positive affect and helps people make sense of what is happening in the context of goals that may be untenable.(41) Our findings show how control is exerted differently in the face of loss, by having more of a focus on the present moment, and by making own decisions about receiving information and care. This has implications for support services, especially in providing options for care, equipment and communication aids.(42) The importance of focusing on the present demonstrates the value of mindfulness-based approaches for psychological interventions. Another novel finding in relation to emotional wellbeing and MND is the importance of self-kindness and self-care. Self-compassion has been associated with adaptive coping strategies and wellbeing other chronic illnesses (43-45) and similar approaches may be useful for pwMND and their caregivers.

Strengths and Limitations

We aimed to recruit a diverse range of participants and succeeded in sampling people with different symptoms of varying severities, particularly people with difficulties speaking. However, we did not manage to recruit many people with mild cognitive impairment. Although we captured the experiences of newly diagnosed people and people who had MND for several years, we could not ascertain if we captured the experiences of people who were at the end stages. Distress and coping strategies used by these groups may be very different and findings need to be applied bearing this in mind.

Conclusion

We provide an in-depth and consolidated understanding of what impacts emotional distress and wellbeing among pwMND and their caregivers. Findings have important implications for psychological interventions, services and professionals that support pwMND and their families. Any communication and support provided for pwMND and their caregivers, needs to pay attention to concepts of hope, control, and compassion.

For peer review only - http://bmjopen.bmj.com/site/about/guidelines.xhtml

Funding statement:

This work was supported by the Motor Neurone Disease Association, UK under Grant [Yardley/May17/891-792].

Competing interest statement:

The authors report no conflict of interest.

Data availability statement:

Anonymised interview transcripts can be made available upon reasonable request from the corresponding author Cathryn Pinto (C.L.Pinto@soton.ac.uk).

Author contributions:

Cathryn Pinto: Main contributor to the design of the study, ethics application, recruitment and data collection, data analysis, reporting and publication of findings.

Dr. Adam Geraghty: Involved in obtaining funding for the study, contributed to the study design, supervised Cathryn during recruitment, data collection and data analysis, and contributed to the reporting and publication of findings.

Prof. Lucy Yardley: Involved in obtaining funding for the study, contributed to the study design and the reporting and publication of findings.

Dr. Laura Dennison: Involved in obtaining funding for the study, contributed to the study design, supervised Cathryn during recruitment, data collection and data analysis, and contributed to the reporting and publication of findings.

For peer review only - http://bmjopen.bmj.com/site/about/guidelines.xhtml

For peer review only

References

1. Moore C, McDermott CJ, Shaw PJ. Clinical aspects of motor neurone disease. Medicine. 2008;36(12):640-5.
2. Clarke DM, McLeod JE, Smith GC, Trauer T, Kissane DW. A comparison of psychosocial and physical functioning in patients with motor neurone disease and metastatic cancer. Journal of palliative care. 2005;21(3):173-9.
3. Goldstein LH, Adamson M, Jeffrey L, Down K, Barby T, Wilson C, et al. The psychological impact of MND on patients and carers. *Journal of the Neurological Sciences.* 1998;160:S114-S21.
4. Hogg K, Goldstein LH, Leigh P. The psychological impact of motor neurone disease. *Psychological medicine.* 1994;24(3):625-32.
5. O'Brien MR, Whitehead B, Jack BA, et al. From symptom onset to a diagnosis of amyotrophic lateral sclerosis/motor neuron disease (ALS/MND): experiences of people with ALS/MND and family carers–a qualitative study. *Amyotrophic lateral sclerosis.* 2011;12(2):97-104.
6. Pavey A, Allen-Collinson J, Pavey T. The lived experience of diagnosis delivery in motor neurone disease: a sociological-phenomenological study. *Sociological research online.* 2013;18(2):36-47.
7. O'Brien MR, Whitehead B, Jack BA, et al. The need for support services for family carers of people with motor neurone disease (MND): views of current and former family caregivers a qualitative study. *Disability and rehabilitation.* 2012;34(3):247-56.
8. Averill AJ, Kasarskis EJ, Segerstrom SC. Psychological health in patients with amyotrophic lateral sclerosis. *Amyotrophic Lateral Sclerosis.* 2007;8(4):243-54.
9. McLeod JE, Clarke DM. A review of psychosocial aspects of motor neurone disease. *Journal of the neurological sciences.* 2007;258(1-2):4-10.
10. Soundy A, Condon N. Patients experiences of maintaining mental well-being and hope within motor neuron disease: a thematic synthesis. *Frontiers in psychology.* 2015;6:606.
11. Goldstein L, Atkins L, Landau S, et al. Longitudinal predictors of psychological distress and self-esteem in people with ALS. *Neurology.* 2006;67(9):1652-8.
12. Bolmsjö I. Existential issues in palliative care: interviews of patients with amyotrophic lateral sclerosis. *Journal of palliative medicine.* 2001;4(4):499-505.
13. Foley G, O'Mahony P, Hardiman O. Perceptions of quality of life in people with ALS: effects of coping and health care. *Amyotrophic lateral sclerosis.* 2007;8(3):164-9.
14. Mistry K, Simpson J. Exploring the transitional process from receiving a diagnosis to living with motor neurone disease. *Psychology & health.* 2013;28(8):939-53.
15. Sakellariou D, Boniface G, Brown P. Experiences of living with motor neurone disease: a review of qualitative research. *Disability and rehabilitation.* 2013;35(21):1765-73.
16. Goldstein LH, Leigh PN. Motor neurone disease: a review of its emotional and cognitive consequences for patients and its impact on carers. *British Journal of Health Psychology.* 1999;4(3):193-208.
17. Pagnini F. Psychological wellbeing and quality of life in amyotrophic lateral sclerosis: a review. *International Journal of Psychology.* 2013;48(3):194-205.
18. Rabkin JG, Albert SM, Rowland LP, et al. How common is depression among ALS caregivers? A longitudinal study. *Amyotrophic Lateral Sclerosis.* 2009;10(5-6):448-55.
19. Cox DL. Perspectives of motor neurone disease. *Clinical rehabilitation.* 1992;6(4):333-9.
20. Brown JB. User, carer and professional experiences of care in motor neurone disease. *Primary Health Care Research & Development.* 2003;4(3):207-17.

For peer review only - http://bmjopen.bmj.com/site/about/guidelines.xhtml

21. Olsson Ozanne AG, Strang S, Persson LI. Quality of life, anxiety and depression in ALS patients and their next of kin. *Journal of clinical nursing.* 2011;20(1-2):283-91.
22. Goldstein L, Atkins L, Leigh P. Correlates of quality of life in people with motor neuron disease (MND). *Amyotrophic Lateral Sclerosis and Other Motor Neuron Disorders.* 2002;3(3):123-9.
23. Matuz T, Birbaumer N, Hautzinger M, et al. Psychosocial adjustment to ALS: a longitudinal study. *Frontiers in psychology.* 2015;6:1197.
24. Gould RL, Coulson MC, Brown RG, et al. Psychotherapy and pharmacotherapy interventions to reduce distress or improve well-being in people with amyotrophic lateral sclerosis: A systematic review. *Amyotrophic Lateral Sclerosis and Frontotemporal Degeneration.* 2015;16(5-6):293-302.
25. Locock L, Mazanderani F, Powell J. Metaphoric language and the articulation of emotions by people affected by motor neurone disease. *Chronic illness.* 2012;8(3):201-13.
26. Whitehead B, O'Brien MR, Jack BA, et al. Experiences of dying, death and bereavement in motor neurone disease: a qualitative study. *Palliative Medicine.* 2012;26(4):368-78.
27. Tong A, Sainsbury P, Craig J. Consolidated criteria for reporting qualitative research (COREQ): a 32-item checklist for interviews and focus groups. *International journal for quality in health care.* 2007;19(6):349-57.
28. Jenkinson C, Fitzpatrick R. Reduced item set for the amyotrophic lateral sclerosis assessment questionnaire: development and validation of the ALSAQ-5. *Journal of Neurology, Neurosurgery & Psychiatry.* 2001;70(1):70-3.
29. Braun V, Clarke V. Using thematic analysis in psychology. *Qualitative research in psychology.* 2006;3(2):77-101.
30. Braun V, Clarke V. Reflecting on reflexive thematic analysis. *Qualitative Research in Sport, Exercise and Health.* 2019;11(4):589-97.
31. Foley G, Timonen V, Hardiman O. Exerting control and adapting to loss in amyotrophic lateral sclerosis. *Social Science & Medicine.* 2014;101:113-9.
32. Hugel H, Grundy N, Rigby S, et al. How does current care practice influence the experience of a new diagnosis of motor neuron disease? A qualitative study of current guidelines-based practice. *Amyotrophic lateral sclerosis.* 2006;7(3):161-6.
33. Oyebode JR, Smith H-J, Morrison K. The personal experience of partners of individuals with motor neuron disease. *Amyotrophic Lateral Sclerosis and Frontotemporal Degeneration.* 2013;14(1):39-43.
34. Galvin M, Corr B, Madden C, et al. Caregiving in ALS–a mixed methods approach to the study of burden. *BMC palliative care.* 2016;15(1):81.
35. Aoun SM, Bentley B, Funk L, et al. A 10-year literature review of family caregiving for motor neurone disease: moving from caregiver burden studies to palliative care interventions. *Palliative medicine.* 2013;27(5):437-46.
36. Larsson BJ, Nordin K, Nygren I. Coping with amyotrophic lateral sclerosis; from diagnosis and during disease progression. *Journal of the neurological sciences.* 2016;361:235-42.
37. Jakobsson Larsson B, Nordin K, Askmark H, et al. Coping strategies among patients with newly diagnosed amyotrophic lateral sclerosis. *Journal of clinical nursing.* 2014;23(21-22):3148-55.
38. Fanos JH, Gelinas DF, Foster RS, et al. Hope in palliative care: from narcissism to self-transcendence in amyotrophic lateral sclerosis. *Journal of Palliative Medicine.* 2008;11(3):470-5.

For peer review only - http://bmjopen.bmj.com/site/about/guidelines.xhtml

39. Foley G, Timonen V, Hardiman O. Understanding psycho-social processes underpinning engagement with services in motor neurone disease: a qualitative study. *Palliative medicine.* 2014;28(4):318-25.
40. Holkham L, Soundy A. The experience of informal caregivers of patients with motor neurone disease: a thematic synthesis. *Palliative & supportive care.* 2018;16(4):487-96.
41. Folkman S, Greer S. Promoting psychological well-being in the face of serious illness: when theory, research and practice inform each other. *Psycho-Oncology: Journal of the Psychological, Social and Behavioral Dimensions of Cancer.* 2000;9(1):11-9.
42. Pavey A, Warren N, Allen-Collinson J. "It gives me my freedom": Technology and responding to bodily limitations in motor neuron disease. *Medical anthropology.* 2015;34(5):442-55.
43. Sirois FM, Molnar DS, Hirsch JK. Self-compassion, stress, and coping in the context of chronic illness. *Self and Identity.* 2015;14(3):334-47.
44. Wren AA, Somers TJ, Wright MA, et al. Self-compassion in patients with persistent musculoskeletal pain: relationship of self-compassion to adjustment to persistent pain. *Journal of pain and symptom management.* 2012;43(4):759-70.
45. Pinto-Gouveia J, Duarte C, Matos M, et al. The protective role of self-compassion in relation to psychopathology symptoms and quality of life in chronic and in cancer patients. *Clinical psychology & psychotherapy.* 2014;21(4):311-23.

For peer review only

For peer review only - http://bmjopen.bmj.com/site/about/guidelines.xhtml

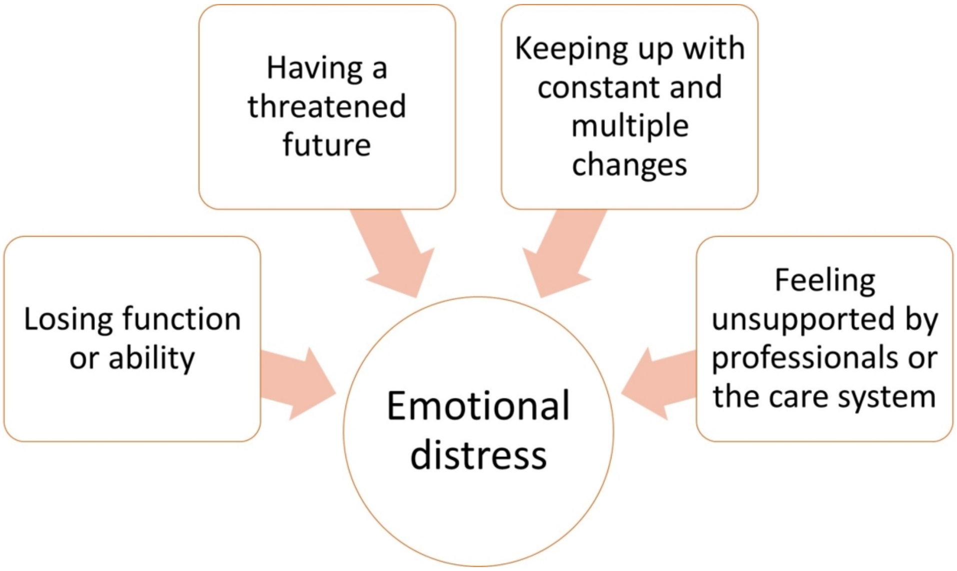

Figure 1. Triggers of emotional distress

167x94mm (300 x 300 DPI)

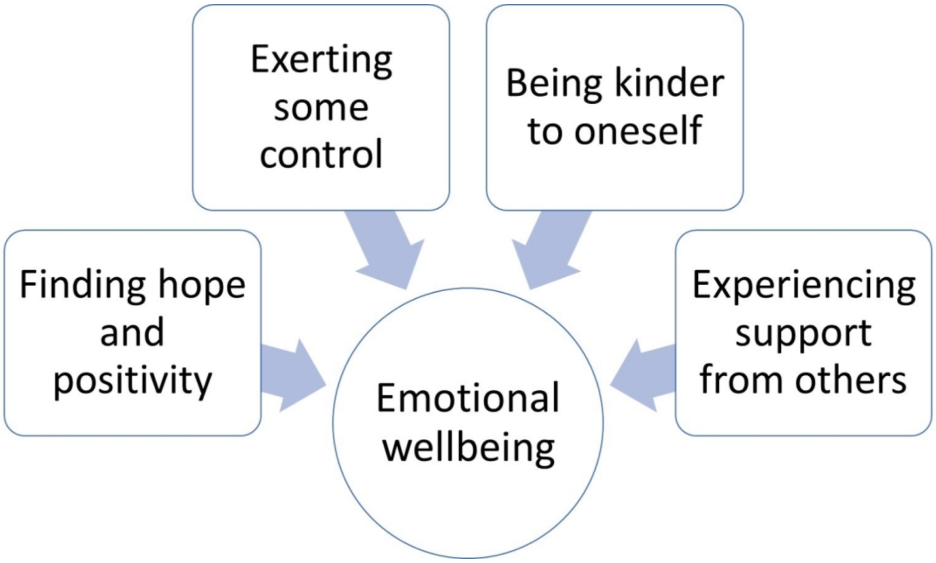

Figure 2. Strategies used to improve emotional wellbeing

427x236mm (96 x 96 DPI)

SUPPLEMENTARY FILE 1

1. Interview topic guide

(1) Can you tell me about your experience with MND?

- wherever the participant would like to start, could be about diagnosis or their experience now.
- Have your thoughts and feelings changed since you were diagnosed or are they the same? (If different, in what way have they changed?)

(2) What's it like to live with MND? Can you tell me a bit more about that?

- What are your main concerns/worries/emotions you face on a day to day basis?
- What are your thoughts and feelings about living with MND?

(3) Can you tell me about how you manage MND on a daily basis? If you find yourself having emotional concerns or worries, do you have any ways of managing them?

(4) Can you tell me about any support you feel you have? Or people/ things/ processes that have helped you?

(5) (In addition to this support) Do you have any thoughts about what else might support you to cope with MND?

For peer review only

- particularly additional support with any emotional concerns/worries that participants have previously mentioned.

For peer review only

SUPPLEMENTARY FILE 2

2. Consolidated criteria for reporting qualitative studies (COREQ):

Tong A, Sainsbury P, Craig J. Consolidated criteria for reporting qualitative research (COREQ): a 32-item checklist for interviews and focus groups. *International Journal for Quality in Health Care*. 2007. Volume 19, Number 6: pp. 349 – 357

For peer review only

**No. Item**	**Guide questions/description**	**Reported on Page #**
**Domain 1: Research team and reflexivity**		
Personal Characteristics		
1. Interviewer/facilitator	Which author/s conducted the interview or focus group?	CP – Methods section
2. Credentials	What were the researcher's credentials? E.g. PhD, MD	PhD student – Methods section, Pg 8
3. Occupation	What was their occupation at the time of the study?	PhD student – Methods section, Pg 8
4. Gender	Was the researcher male or female?	Female
5. Experience and training	What experience or training did the researcher have?	Methods section – Pg 8-9
Relationship with participants		
6. Relationship established	Was a relationship established prior to study commencement?	No
7. Participant knowledge of the interviewer	What did the participants know about the researcher? e.g. personal goals, reasons for doing the research	Participants knew that the researcher was a PhD student at the University of Southampton, doing her PhD on emotional distress and wellbeing in Motor Neurone Disease (MND). This information was described on the participant information sheets and

		study adverts.
8. Interviewer characteristics	What characteristics were reported about the interviewer/facilitator? e.g. Bias, assumptions, reasons and interests in the research topic	The interviewer did not have much prior knowledge of MND, but had previously worked with and interviewed people with Parkinson's disease and people receiving palliative care.
**Domain 2: study design**		
Theoretical framework		
9. Methodological orientation and Theory	What methodological orientation was stated to underpin the study? e.g. grounded theory, discourse analysis, ethnography, phenomenology, content analysis	The study used methods (in-depth semi-structured interviews and reflexive thematic analysis) in line with a phenomenological orientation.
Participant selection		
10. Sampling	How were participants selected? e.g. purposive, convenience, consecutive, snowball	Purposive sampling – Methods section, Pg 6
11. Method of approach	How were participants approached? e.g. face-to-face, telephone, mail, email	Methods section – Pg 8
12. Sample size	How many participants were in the study?	Methods section – Pg 6
13. Non-participation	How many people refused to participate or dropped out? Reasons?	Participants contacted the research if they wanted to take part in an interview. No one dropped out.
Setting		
14. Setting of data collection	Where was the data collected? e.g. home, clinic, workplace	Methods section – Pg 8
15. Presence of non-participants	Was anyone else present besides the participants and researchers?	No
16. Description of sample	What are the important characteristics of the sample? e.g. demographic data, date	Methods section – Pgs 6-7
Data collection		
17. Interview guide	Were questions, prompts, guides	Methods section, Pg 8 and

For peer review only

	provided by the authors? Was it pilot tested?	supplementary file 1
18. Repeat interviews	Were repeat interviews carried out? If yes, how many?	N/A
19. Audio/visual recording	Did the research use audio or visual recording to collect the data?	Methods section – Pg 8
20. Field notes	Were field notes made during and/or after the interview or focus group?	Field notes were made after each interview.
21. Duration	What was the duration of the interviews or focus group?	Average duration reported – Methods section, Pg 8
22. Data saturation	Was data saturation discussed?	No. We were not aiming for theoretical data saturation, but rather to look at patterns of experience across participants with diverse characteristics.
23. Transcripts returned	Were transcripts returned to participants for comment and/or correction?	No
**Domain 3: analysis and findings**		
Data analysis		
24. Number of data coders	How many data coders coded the data?	One – Methods section, Pgs 8-9
25. Description of the coding tree	Did authors provide a description of the coding tree?	N/A
26. Derivation of themes	Were themes identified in advance or derived from the data?	Inductive thematic analysis – Methods section, Pg 8-9
27. Software	What software, if applicable, was used to manage the data?	NVivo
28. Participant checking	Did participants provide feedback on the findings?	A summary of findings was sent to participants and some participants reported that the overall findings resonated with their experience.
Reporting		
29. Quotations presented	Were participant quotations	Yes, Results section

For peer review only

	presented to illustrate the themes/findings? Was each quotation identified? e.g. participant number	
30. Data and findings consistent	Was there consistency between the data presented and the findings?	Yes, illustrative quotations have been provided for each theme and sub-theme – Results section
31. Clarity of major themes	Were major themes clearly presented in the findings?	Results section and presented in Figures 1 & 2
32. Clarity of minor themes	Is there a description of diverse cases or discussion of minor themes?	Description of diverse cases embedded within the explanation of themes – Results section

For peer review only

BMJ Open

Emotional distress and wellbeing: a thematic analysis of interviews with people with Motor Neurone Disease (MND) and their family caregivers

Journal:	*BMJ Open*
Manuscript ID	bmjopen-2020-044724.R1
Article Type:	Original research
Date Submitted by the Author:	23-Dec-2020
Complete List of Authors:	Pinto, Cathryn; University of Southampton, Psychology<br>Geraghty, Adam; University of Southampton, Primary Care and Population Sciences<br>Yardley, Lucy; University of Southampton, Psychology; University of Bristol, School of Health Sciences<br>Dennison, Laura; University of Southampton
**Primary Subject Heading**:	Neurology
Secondary Subject Heading:	Qualitative research, Mental health
Keywords:	Motor neurone disease < NEUROLOGY, Depression & mood disorders < PSYCHIATRY, Anxiety disorders < PSYCHIATRY, QUALITATIVE RESEARCH

SCHOLARONE™
Manuscripts

I, the Submitting Author has the right to grant and does grant on behalf of all authors of the Work (as defined in the below author licence), an exclusive licence and/or a non-exclusive licence for contributions from authors who are: i) UK Crown employees; ii) where BMJ has agreed a CC-BY licence shall apply, and/or iii) in accordance with the terms applicable for US Federal Government officers or employees acting as part of their official duties; on a worldwide, perpetual, irrevocable, royalty-free basis to BMJ Publishing Group Ltd ("BMJ") its licensees and where the relevant Journal is co-owned by BMJ to the co-owners of the Journal, to publish the Work in this journal and any other BMJ products and to exploit all rights, as set out in our licence.

The Submitting Author accepts and understands that any supply made under these terms is made by BMJ to the Submitting Author unless you are acting as an employee on behalf of your employer or a postgraduate student of an affiliated institution which is paying any applicable article publishing charge ("APC") for Open Access articles. Where the Submitting Author wishes to make the Work available on an Open Access basis (and intends to pay the relevant APC), the terms of reuse of such Open Access shall be governed by a Creative Commons licence – details of these licences and which Creative Commons licence will apply to this Work are set out in our licence referred to above.

Other than as permitted in any relevant BMJ Author's Self Archiving Policies, I confirm this Work has not been accepted for publication elsewhere, is not being considered for publication elsewhere and does not duplicate material already published. I confirm all authors consent to publication of this Work and authorise the granting of this licence.

For peer review only

Emotional distress and wellbeing: a thematic analysis of interviews with people with Motor Neurone Disease (MND) and their family caregivers

Cathryn Pinto,[a]* Adam W A Geraghty[b], Lucy Yardley[a,c] and Laura Dennison[a]

[a]*Department of psychology, University of Southampton, Southampton, UK;*

[b]*Primary care, population sciences and medical education, University of Southampton, Southampton, UK;*

[c]*School of psychological science, University of Bristol, Bristol, UK*

*Corresponding author: Cathryn Pinto, University of Southampton, B44 Highfield campus, Southampton SO17 1BJ Email: C.L.Pinto@soton.ac.uk

Word count: 4339 (including quotes and table)

For peer review only - http://bmjopen.bmj.com/site/about/guidelines.xhtml

Abstract

Objective: We aimed to get an in-depth understanding of the emotions experienced by people with Motor Neurone Disease (MND) and their caregivers and to explore what impacts emotional distress and wellbeing.

Design: Qualitative study using semi-structured interviews with people with MND and caregivers.

Setting: Participants were recruited from across the UK and took part in interviews conducted either face-to-face, by telephone or email to accommodate for varying levels of disability.

Participants: 25 people with MND and 10 caregivers took part. Participants were purposively sampled based on their MND diagnosis, symptoms and time since diagnosis.

Data analysis: Data were analysed using inductive reflexive thematic analysis.

Results: Eight broad themes were generated (20 subthemes). Participants described the emotional distress of losing physical function and having a threatened future because of poor prognosis. Keeping up with constant changes in symptoms and feeling unsupported by the healthcare system added to emotional distress. Finding hope and positivity, exerting some control, being kinder to oneself, and experiencing support from others were helpful strategies for emotional wellbeing.

Conclusion: We provide an in-depth understanding of what impacts emotional distress and wellbeing and discuss implications for psychological interventions for people with MND and their family. Any communication and support provided for people with MND and their caregivers, needs to pay attention to concepts of hope, control, and compassion.

For peer review only

Keywords: Motor neuron disease; Amyotrophic lateral sclerosis; emotional distress; wellbeing; depression; qualitative research

Article summary (Strengths and Limitations):

- Purposive sampling and flexible recruitment strategies were used to capture experiences of people with MND with a range of symptoms, and at different lengths of time since diagnosis.
- Participants with mild cognitive impairment were underrepresented in the sample.
- Conducting email interviews ensured we included the experiences of people with speech difficulties; however there were some methodological difficulties in using prompts and asking follow up questions.
- Study design did not allow us to see changes in emotional of distress and wellbeing over time, as symptoms change and people find new ways to adjust to changes.

For peer review only - http://bmjopen.bmj.com/site/about/guidelines.xhtml

INTRODUCTION

Motor neurone disease (MND) is a neurodegenerative disease, which results in declining physical function and has a very poor prognosis.(1) Many people with MND (pwMND) experience depression.(2-4) Anxiety is prevalent around the time of diagnosis(5, 6) and during the final stages.(7) PwMND also experience other forms of emotional distress besides anxiety and depression including feelings of hopelessness(8-10) and demoralization.(2) Emotions are also affected by low self-esteem,(4, 11) end of life concerns,(8) faith/existential concerns,(12, 13) sense of loss,(13, 14) and changes in identity, roles and relationships.(11, 13, 15) We have used the term 'emotional distress' to refer to this broad range of negative emotional states.

Family members or caregivers of pwMND often struggle with the emotional impact and burden of the disease, and have high rates of psychological morbidity.(16-18) Studies with caregivers have described the strain from caring tasks, from having to make changes to their own lives and not having time for themselves.(19-21)

Research has attempted to understand more about this experience of distress and factors that might explain emotional distress or protect against it. Results from these studies show that psychosocial factors like coping strategies and social support, are more strongly related to wellbeing and quality of life than demographic and clinical factors.(11, 22, 23) Qualitative research can be useful for understanding emotional distress and wellbeing because it allows participants to express their own understandings and experiences without being limited to concepts pre-determined by the researcher. Qualitative studies have largely explored the experience and impact of living with MND,(10, 13, 19, 24) which have added to our understanding of distress in MND. However, most existing qualitative studies have not specifically focused on the experience of emotional distress and wellbeing. One qualitative

For peer review only - http://bmjopen.bmj.com/site/about/guidelines.xhtml

study examined the use of metaphors to express emotions,(25) and a small number have focused on emotions at specific time periods (e.g. during diagnosis or the final stages).(6, 14, 26) Emotional distress at other time points and other issues related to distress and wellbeing are largely unexplored.

This study aims to fill these gaps and build on our understanding of emotional distress and wellbeing in MND. More importantly, we aim to hear from people whose experiences are not well represented in research, including people with speech difficulties and cognitive impairment. Recent articles have highlighted that there are few effective psychological interventions to improve wellbeing,(27) and more interventions are needed.(17) This study is part of a project to develop an intervention to improve emotional wellbeing among pwMND and caregivers.

In this current study, we aim to explore the emotional impact of living with MND, and what impacts experiences of emotional distress and wellbeing among pwMND and their family caregivers.

For peer review only - http://bmjopen.bmj.com/site/about/guidelines.xhtml

METHODS

Design

Qualitative study using in-depth semi-structured interviews and reflexive thematic analysis, in line with an interpretivist approach. The paper is reported in accordance with the Consolidated criteria for reporting qualitative research (COREQ) (see Supplementary file 2).(28)

Participants

We aimed to recruit 20-30 pwMND and used purposive sampling to represent people with difficulties with movement, speech and cognition, and different lengths of time since diagnosis. Caregiver participants had fewer sampling criteria (age, gender), therefore we aimed to recruit 10-15 caregivers.

Eligibility: Participants were above 18 years of age, had an MND diagnosis, and had mental capacity to consider participation in the study (assessed by the researcher through correspondence about the study). We included participants who self-reported difficulties with cognition, but had mental capacity to give informed consent, as the views of pwMND who have cognitive impairment have been underrepresented in previous research. We included caregivers above 18 years of age, both current and recently bereaved (bereavement within 1 year from the time of the interview).

Data collection

Participants were recruited through a UK charity that supports pwMND and their families (Motor Neurone Disease Association). Study information was circulated via the charity's website, newsletters, social media outlets and local support groups. People willing to take part contacted CP, who screened for eligibility and provided further details about the interview.

For peer review only - http://bmjopen.bmj.com/site/about/guidelines.xhtml

Before each interview, participants gave written informed consent and filled a demographic/clinical details form. This process was completed either in-person, by post or email, based on the interview mode. CP, who has training and experience in conducting interviews for qualitative research, conducted all interviews. Interview mode was flexible (face-to-face, email or phone) to account for various levels of disability. Face-to-face interviews were conducted in a place convenient to participants, usually at home or a hospice. Where two members of a couple had both consented to participate participants were interviewed separately where possible, but jointly where this was requested. The interview topic guide was developed iteratively by CP, LD, AG and patient and public involvement members. In line with an interpretivist approach, questions were broad and open-ended; follow-up questions were led by participants' responses. The final interview topic guide (Supplementary file 1) covered questions about people's experiences living with MND, with a focus on their thoughts and feelings and coping with emotional concerns.

Data analysis

Face-to-face and phone interviews were audio recorded and transcribed, and all interviews were anonymized. Field notes were completed after each interview, reflecting on participants' responses, interview method and procedure. Data were analyzed using reflexive thematic analysis,(29, 30) as this was a flexible method that suited the research question, helped us identify common patterns across participants' experiences and allowed us to look for underlying meaning behind experiences of emotional distress and wellbeing. In line with an interpretivist approach, we used an inductive approach to data analysis, and included convergent and divergent cases in theme development. The analysis was mainly conducted by CP, who first familiarised herself with interview transcripts, and used Nvivo 12 to code

For peer review only - http://bmjopen.bmj.com/site/about/guidelines.xhtml

the data inductively. The codes went through several iterations as new interviews were coded and notes were made about interesting features of the data. Similar codes about emotional distress and emotional wellbeing were grouped together and candidate themes were generated. Themes were then reviewed, theme names were revised and findings written up. This was an iterative process whereby descriptions were clarified and overlap between themes identified. CP had regular meetings with qualitative research experts LD and AG who helped refine codes and themes. A lay summary of the findings was sent to all participants.

Ethics

We obtained ethics from the University of Southampton ethics committee (ERGOII-46996). Written informed consent was obtained for all participants. Participants were debriefed and steps were taken following each interview to ensure participants were not distressed.

Patient and Public Involvement (PPI)

Three PPI members (one person with MND and 2 former caregivers) contributed to this study. They were involved in the recruitment stage to help identify potential participants. They also helped pilot the interview topic guide, refine interview questions and trial the data collection procedure to ensure that it was not burdensome. Some PPI members looked over early drafts of the findings and gave feedback for the reporting of the results.

RESULTS

Participants were 25 pwMND and 10 family caregivers (see Table 1). 29 interviews were conducted in total; 6 joint interviews and 23 one-to-one interviews with either the pwMND or

For peer review only

caregiver. 14 interviews were conducted face-to-face, 8 via telephone, and 7 via email. Interviews lasted an average of 39.6 minutes.

Table 1: Demographic and clinical details of the sample

Characteristic	People with MND (n=25)	Caregivers (n=10)
**Age (range 39-80)**		
35-50 years	3	0
51-65 years	12	4
66-80 years	10	6
**Gender**		
Male	15	5
Female	10	5
**Relationship to person with MND**		
Spouse/partner		10
**Diagnosis**		
ALS limb	18	
ALS bulbar	1	
Primary Lateral Sclerosis	4	
Progressive Bulbar Palsy	1	
Progressive Muscular Atrophy	1	
**Time since diagnosis (range 2 months-17 years)**		
Less than 1 year	8	
1-3 years	6	

For peer review only
For peer review only - http://bmjopen.bmj.com/site/about/guidelines.xhtml

3-5 years	7
More than 5 years	4
**Difficulties reported often/always *(ALSAQ-5 scale)**	
Difficult to stand up	16
Difficult to use arms and hands	16
Difficulty eating solid food	4
Speech is not easy to understand	9
Feeling hopeless about the future	3
**Self-reported concerns about cognitive ability**	2

*Amyotrophic Lateral Sclerosis Assessment Questionnaire (ALSAQ-5) is a patient self-report questionnaire used to briefly measure the impact of ALS/MND on patients.(31)

We developed 8 themes and 20 subthemes. The first four themes relate to triggers of emotional distress (Fig 1); the remaining four themes capture strategies to improve emotional wellbeing (Fig 2). All themes were present in some degree and form in both patient and caregiver interviews; where differences between these groups were apparent, they are described.

[Insert Fig 1 here]

Triggers of emotional distress

Participants described 4 main triggers of emotional distress and how they had an impact on emotions (themes are numbered, *subthemes in italics*).

For peer review only

(1) Losing function or ability

Most participants described how losing function or ability was distressing because of a *reduced sense of autonomy and control.* PwMND spoke about feelings of anger, frustration, sadness or grief at not being able to do the things they wanted to, at losing their independence and relying on others for help. Caregivers expressed similar emotions at seeing their family member lose capability and independence.

> "Hobbies that I had, I can't pursue any of them because I just don't have any capability enough in my arms now to do that. So, I think mentally I've seen a dip in the last six months into a much more kind of negative and angry stage really." (Person with MND, P13)

Losing function led to *changes in self-identity*, specifically those of becoming a 'patient' and 'carer'. These changes, especially losing one's voice which is strongly linked to identity, were experienced as distressing.

> "Losing mobility gradually was bad enough… but losing my voice is much more dreadful. It's taking away part of my personality. I can no longer express myself properly." (Person with MND, P21)

Losing function or ability sometimes led to *changes in relationships and interactions*. PwMND spoke about negative feelings of becoming a burden for their partner/spouse. Both patient and caregiver participants spoke about how having MND sometimes changed or limited interactions between family members.

For peer review only - http://bmjopen.bmj.com/site/about/guidelines.xhtml

> "It's emotionally difficult not being able to physically help my son or my parents and siblings and friends. Not being able to hold and play with my nephew." (Person with MND, P26)

Participants also had *practical concerns or worries about managing tasks* as a result of losing more ability and function.

> "My main worry at the moment really is moving him from there to there because his knees and lower legs are getting weaker and I've got that [points to hoist] to move him." (Caregiver, C03)

(2) Having a threatened future

The short prognosis that accompanies a diagnosis of MND led to feelings of anger, disappointment, sadness and feelings of being robbed/cheated of a future (*threat to life narrative and future plans*).

> "But I'm having to accept I might have a lonely old age. Disappointment, more than anything else.' (Caregiver, C27)

Participants also described emotional distress when *anticipating future symptoms or disease progression*. Many things triggered worry about the future including researching MND online, meeting other pwMND, discussions with healthcare professionals and going through symptom checklists. Some participants also spoke about experiencing such worries about the future even if symptoms had not progressed or were relatively stable. This was true

For peer review only - http://bmjopen.bmj.com/site/about/guidelines.xhtml

particularly just after diagnosis, where worries about the future led to feelings of depression, low mood and withdrawal from others.

> “Even though physically, say that first month afterwards, there was almost no change but my mind’s thinking quite morose almost. You almost think of death at that point... It’s not even anger at that stage, it’s just hopelessness at that point because you’re just thinking oh that is it isn’t it? It’s all over.” (Person with MND, P10)

The *uncertainty of the disease progression* also caused emotional distress among both patients and caregivers, because of being unable to manage symptoms or plan for the future.

> “I think the biggest thing about this is that it’s such an unknown. Because everybody is different and, who knows how much, how long this disease is going to affect you. And the uncertainty, for me, it’s very hard to cope with.’ (Caregiver, C15)

(3) Keeping up with constant and multiple changes

Participants discussed how the *timing and relentless nature of changes* in symptoms was difficult physically and emotionally. As time went on, for some it became easier to fall into a care routine. However, if deterioration happened quickly, both patient and caregivers expressed that it was difficult to cope and left them feeling tired, not in control and in need of respite.

For peer review only - http://bmjopen.bmj.com/site/about/guidelines.xhtml

> "If something's difficult, we find a way round it. And by the time we've found a way round it, things have moved on again, and it doesn't work. So that's very frustrating. And a bit… not depressing… soul destroying, because we're always playing catch up." (Person with MND, P24)

Sometimes physical symptoms necessitated *changes in many areas of life.* This included changes to their home, work, social and leisure activities. Both pwMND and caregivers spoke about the effort involved in either living with MND or looking after someone with MND. This sometimes affected thoughts and mood negatively including feelings of frustration, anxiety, tiredness or strain/burden. For example, C03, a caregiver, recalled being upset and needing someone to talk to due to exhaustion from physically moving her spouse; "I used to get so tired that sometimes when he had gone to bed in the evening I did just feel like standing there crying."

(4) Feeling unsupported by professionals or the care system

Participants reported feeling distressed when healthcare professionals or the care system did not support their needs. *Infrequent or inefficient care provision* left people feeling abandoned and left on their own to deal with MND. This was felt particularly around diagnosis if there was a gap between appointments or no follow up support, leaving pwMND and families on their own to cope with the shock, questions and feelings of hopelessness that often accompanied receiving an MND diagnosis.

> "I felt completely unsupported by the neurologist or the health service, we were really just left on our own, completely in the dark as to what was gonna happen... So, it was a very, very distressing time." (Person with MND, P14)

For peer review only - http://bmjopen.bmj.com/site/about/guidelines.xhtml

Poor coordination between different professionals/organizations, difficulty accessing equipment and financial benefits also triggered feelings of frustration, injustice and being 'let down' by the system, especially if participants didn't know where to go for support or if they had to struggle/fight to get the right support.

Poor or insensitive communication from healthcare professionals added to emotional distress. In particular, participants described upset when professionals were not sensitive to individual's preferences for receiving information, did not show empathy for the devastating impact of the disease/diagnosis for pwMND and caregivers, and when only decline was highlighted without presenting avenues for support.

> "Our life was shattered in 2 minutes. And I felt that was cruel, very very cruel [pause]. There should be a way of discussing it with you and saying "well look, you have such and such a condition, but hey, this is what we can do." Not just you've got this and throw you out, and so we had to find our own way round this problem." (Caregiver, C01)

[Insert Fig. 2 here]

Strategies used to improve emotional wellbeing

Participants described strategies they used to improve emotional wellbeing (themes are numbered; *subthemes in italics*).

For peer review only - http://bmjopen.bmj.com/site/about/guidelines.xhtml

(1) Finding hope and positivity

Despite the many losses, participants actively found ways to introduce hope and positivity. For many people, acceptance of MND was a gradual, and even once there was acceptance, an attitude of not giving up on life was important (*acceptance and not giving up)*.

> "I always wanna think I've got options, got somewhere to go because at the point where you think 'well I've got nowhere left to go with this', that's when you may sort of deteriorate and let it get the best of you. But I think if you say 'right there's this trial, this might happen, I'll go and see a physio…and just see if that helps me." (Person with MND, P18)

For some, not giving up included hoping for a cure or trying alternative therapies. For others, not giving up included finding solutions to make life easier, and not letting MND define them as they continued normal and valued activities. This strategy was endorsed mainly by pwMND.

> "not giving up, not allowing it to put you away in a corner somewhere, waiting for the inevitable…not allowing that to define what you do with whatever time you have left." (Person with MND, P06)

Despite severe losses of function, many participants continued *doing activities that they enjoyed and that were meaningful*. Having things to look forward to in the immediate future added to feelings of hope.

> "Key thing is to have lots of little goals, lots of little hopes of things to do, normalities, weekends away, holidays but not in the far future. Everything's in a couple of weeks, 2-3 weeks, something to look forward to… it keeps the

For peer review only - http://bmjopen.bmj.com/site/about/guidelines.xhtml

> mind occupied to know that there is something coming up." (Person with MND, P10)

Most participants tried to *focus on the positive aspects of life*. This strategy became difficult in the context of increasing loss, but participants still stressed that it was important.

> "I'm very much sort of an optimist. You can't…you know, because it's horrible we know that. But we just make [name of pwMND] as comfortable as possible. You've got to be positive, otherwise you just sink and go under." (Caregiver, C07)

To stay positive and deal with negative emotions, participants tried to reframe difficult situations by focusing on what they still can do, using humour, feeling grateful for symptoms they did not have or feeling lucky because of their circumstances or because of the support they received.

(2) Exerting some control

When MND took some control away, participants found other ways to exert control over their lives. Although a sense of control was important for most participants, they exerted this control in very different ways. One way was by *focusing on the present moment* and making the most of what they could currently do. This strategy was adopted mainly by pwMND; it stopped them from worrying about the future or feeling sad for what they could no longer do, and instead had a more positive impact on emotions.

For peer review only - http://bmjopen.bmj.com/site/about/guidelines.xhtml

> "I realised there's absolutely no point in dwelling on the past because there's absolutely nothing I can do to get that back… and there's no point in worrying about the future because there's absolutely nothing I can do about it. So, what I kind of taught myself to do is try to live in here and now because I do have some control over that." (Person with MND, P04)

Some participants described how it was helpful to exert *control over decisions about their care,* organizing or planning care and taking a more active problem-solving approach. This gave people a sense of empowerment and sense of autonomy, when they would otherwise be struggling to cope.

> "I hate the fact that I can't talk. Although I can talk, but it's a bit drained and doesn't sound like me. But I've done voice banking and I think you've got to be a step ahead." (Person with MND, P19)

> 'they're [professionals] pretty good at picking up what might be the next thing that's necessary or how tired I'm looking and dropping in a suggestion here and there about what you might change, what you might need next… in terms of future proofing, so you're on top of a problem and not floundering about solving it.' (Caregiver, C05)

However, some people exerted control by avoiding interactions or information that might remind them of future symptoms or deterioration and associated negative emotions.

For peer review only - http://bmjopen.bmj.com/site/about/guidelines.xhtml

> "Everyone's different, aren't they? And their MND is different. Some people like to know the ins and out, but I don't know, I like to bury my head in the sand. Because I know that I would sit and worry about it all the time. And I don't want to." (Person with MND, P24)

(3) Being kinder to oneself

This strategy was mentioned by both pwMND and caregivers but for different kinds of tasks. Living with MND was often described as overwhelming and participants expressed the need for *taking a break* from MND. Caregivers spoke about the need to take a physical break from MND care; while pwMND spoke about taking a break from MND by doing normal activities and not thinking about MND.

> "I'll have days where I deliberately avoid looking at anything and trying just to have days or a couple of days where I don't think about MND." (Person with MND, P18)

Some participants also expressed the need to give themselves *time and space to adjust to changes* in symptoms. This was done by allowing themselves space to vent if they felt overwhelmed, pacing their activities, lowering expectations, modifying how tasks were done, or asking for help.

> "Keep doing the things you enjoy, just lower your expectations and standards so you achieve and don't feel disappointed." (Person with MND, P21)

(4) Experiencing support from others

For peer review only - http://bmjopen.bmj.com/site/about/guidelines.xhtml

Support from people in a similar situation Participants valued support from other people with MND and caregivers because they felt truly understood, less alone, and learned of practical solutions to cope with disability. Some participants found it difficult to meet other people with MND, but once they felt ready to do so, they found this to be a positive experience.

> "When you're still ambulant and not in a wheelchair, it's a bit scary meeting other people that are a little bit further along the road. But actually we gained a lot of strength from those people." (Caregiver, C05)

Sensitive and supportive communication from healthcare professionals gave pwMND and families some comfort. This included providing information based on the patient and families' readiness, communicating information in a sensitive and empathic manner, and focusing on what can be done in terms of care.

> "She delivered the diagnosis, she didn't give me a prognosis, how long I might have, which I was grateful for in a way because we didn't ask for it. She just said it's a life-limiting disease." (Person with MND, P06)

Timely provision and availability of support was also important with changing symptoms and needs. Having a multidisciplinary care team and specialist support who knew about MND specific problems and solutions had a positive impact on pwMND and families. Participants valued well-coordinated care and professionals who anticipated their support needs.

For peer review only - http://bmjopen.bmj.com/site/about/guidelines.xhtml

"I would also mention my occupational therapist who has been brilliant at assessing my needs and getting in equipment quickly, usually just before they were needed. This has given me more confidence in the care and support I am given." (Person with MND, P22)

For peer review only

For peer review only - http://bmjopen.bmj.com/site/about/guidelines.xhtml

DISCUSSION

This study provides an in-depth understanding of the experience of emotional distress and emotional wellbeing among people with MND and their caregivers. Our findings come from a diverse sample, thereby highlighting key triggers of distress and coping strategies used by people with different abilities, symptoms and at different stages of having MND. PwMND and caregivers use coping strategies differently but still rely on similar concepts (hope, positivity, control, self-kindness, social support) to improve emotional wellbeing.

Some of our findings are in line with previous MND research, such as the distress caused by loss of ability and threats to the future.(10, 14, 15, 32) We also know that distress can be caused through not receiving appropriate professional support.(6, 7, 26, 33) We highlight the emotional distress triggered by multiple and constant changes brought about by disease progression and constantly 'playing catch up.' This is a new finding in terms of emotional distress for pwMND, but has been described in the literature around caregiver experience;(34-36). This finding is important for intervention development, and we need to ensure that interventions are perceived as manageable, not burdensome.

Coping strategies are important for emotional wellbeing,(23, 37, 38) and hope and control are particularly important for MND.(10, 32, 39-41) Soundy and Condon (2015) show how concepts of hope and control are linked to each other, and can affect mental wellbeing in MND.(10) Our findings highlight the complexities of using hope and control in a disease where this is constantly being threatened, and provide examples of how pwMND and caregivers use hope and control to improve wellbeing. For example, we found that many people use meaning-based coping strategies (finding hope and positivity in the face of difficult circumstances and reappraising them in a positive way), which generates positive

For peer review only - http://bmjopen.bmj.com/site/about/guidelines.xhtml

affect and helps people make sense of what is happening in the context of goals that may be untenable.(42) Our findings also show how control is exerted differently in the face of loss, by focusing more on the present moment, or by making decisions about receiving information and care. This has implications for support services, especially in providing options for care, equipment and communication aids.(43) With MND, thinking about the future can be distressing, but not planning for the future and not having the right support to manage symptoms can also be distressing. This can be complicated for services to tailor support based on individual's level of readiness, but support, including psychological support, needs to be flexible to meet this variability in need.(44) Our findings also point to the potential usefulness of mindfulness-based and compassion-focused therapy for pwMND and their caregivers.

Strengths and Limitations

We aimed to recruit a diverse range of participants and succeeded in sampling people with different symptoms of varying severities, particularly people with difficulties speaking. However, we did not manage to recruit many people with mild cognitive impairment and could have benefitted from a more targeted sampling approach. Distress and coping strategies may be very different for people with cognitive impairment and findings need to be applied bearing this in mind.

Conclusion

We provide an in-depth and consolidated understanding of what impacts emotional distress and wellbeing among pwMND and their caregivers. Findings have important implications for psychological interventions, services and professionals that support pwMND and their families. Any communication and support provided for pwMND and their caregivers, needs to pay attention to concepts of hope, control, and compassion.

For peer review only - http://bmjopen.bmj.com/site/about/guidelines.xhtml

Funding statement:

This work was supported by the Motor Neurone Disease Association, UK under Grant [Yardley/May17/891-792].

Competing interest statement:

The authors report no conflict of interest.

Data availability statement:

Anonymised interview transcripts can be made available upon reasonable request from the corresponding author Cathryn Pinto (C.L.Pinto@soton.ac.uk).

Author contributions:

Cathryn Pinto: Main contributor to the design of the study, ethics application, recruitment and data collection, data analysis, reporting and publication of findings.

Dr. Adam Geraghty: Involved in obtaining funding for the study, contributed to the study design, supervised Cathryn during recruitment, data collection and data analysis, and contributed to the reporting and publication of findings.

Prof. Lucy Yardley: Involved in obtaining funding for the study, contributed to the study design and the reporting and publication of findings.

Dr. Laura Dennison: Involved in obtaining funding for the study, contributed to the study design, supervised Cathryn during recruitment, data collection and data analysis, and contributed to the reporting and publication of findings.

For peer review only - http://bmjopen.bmj.com/site/about/guidelines.xhtml

References

1. Moore C, McDermott CJ, Shaw PJ. Clinical aspects of motor neurone disease. Medicine. 2008;36(12):640-5.
2. Clarke DM, McLeod JE, Smith GC, Trauer T, Kissane DW. A comparison of psychosocial and physical functioning in patients with motor neurone disease and metastatic cancer. Journal of palliative care. 2005;21(3):173-9.
3. Goldstein LH, Adamson M, Jeffrey L, Down K, Barby T, Wilson C, et al. The psychological impact of MND on patients and carers. Journal of the Neurological Sciences. 1998;160:S114-S21.
4. Hogg K, Goldstein LH, Leigh P. The psychological impact of motor neurone disease. Psychological medicine. 1994;24(3):625-32.
5. O'Brien MR, Whitehead B, Jack BA, Mitchell JD. From symptom onset to a diagnosis of amyotrophic lateral sclerosis/motor neuron disease (ALS/MND): experiences of people with ALS/MND and family carers–a qualitative study. Amyotrophic lateral sclerosis. 2011;12(2):97-104.
6. Pavey A, Allen-Collinson J, Pavey T. The lived experience of diagnosis delivery in motor neurone disease: a sociological-phenomenological study. Sociological research online. 2013;18(2):36-47.
7. O'Brien MR, Whitehead B, Jack BA, Mitchell JD. The need for support services for family carers of people with motor neurone disease (MND): views of current and former family caregivers a qualitative study. Disability and rehabilitation. 2012;34(3):247-56.
8. Averill AJ, Kasarskis EJ, Segerstrom SC. Psychological health in patients with amyotrophic lateral sclerosis. Amyotrophic Lateral Sclerosis. 2007;8(4):243-54.
9. McLeod JE, Clarke DM. A review of psychosocial aspects of motor neurone disease. Journal of the neurological sciences. 2007;258(1-2):4-10.
10. Soundy A, Condon N. Patients experiences of maintaining mental well-being and hope within motor neuron disease: a thematic synthesis. Frontiers in psychology. 2015;6:606.
11. Goldstein L, Atkins L, Landau S, Brown R, Leigh P. Longitudinal predictors of psychological distress and self-esteem in people with ALS. Neurology. 2006;67(9):1652-8.
12. Bolmsjö I. Existential issues in palliative care: interviews of patients with amyotrophic lateral sclerosis. Journal of palliative medicine. 2001;4(4):499-505.
13. Foley G, O'Mahony P, Hardiman O. Perceptions of quality of life in people with ALS: effects of coping and health care. Amyotrophic lateral sclerosis. 2007;8(3):164-9.
14. Mistry K, Simpson J. Exploring the transitional process from receiving a diagnosis to living with motor neurone disease. Psychology & health. 2013;28(8):939-53.
15. Sakellariou D, Boniface G, Brown P. Experiences of living with motor neurone disease: a review of qualitative research. Disability and rehabilitation. 2013;35(21):1765-73.
16. Goldstein LH, Leigh PN. Motor neurone disease: a review of its emotional and cognitive consequences for patients and its impact on carers. British Journal of Health Psychology. 1999;4(3):193-208.
17. Pagnini F. Psychological wellbeing and quality of life in amyotrophic lateral sclerosis: a review. International Journal of Psychology. 2013;48(3):194-205.
18. Rabkin JG, Albert SM, Rowland LP, Mitsumoto H. How common is depression among ALS caregivers? A longitudinal study. Amyotrophic Lateral Sclerosis. 2009;10(5-6):448-55.
19. Cox DL. Perspectives of motor neurone disease. Clinical rehabilitation. 1992;6(4):333-9.
20. Brown JB. User, carer and professional experiences of care in motor neurone disease. Primary Health Care Research & Development. 2003;4(3):207-17.

For peer review only - http://bmjopen.bmj.com/site/about/guidelines.xhtml

For peer review only

21. Olsson Ozanne AG, Strang S, Persson LI. Quality of life, anxiety and depression in ALS patients and their next of kin. Journal of clinical nursing. 2011;20(1-2):283-91.
22. Goldstein L, Atkins L, Leigh P. Correlates of quality of life in people with motor neuron disease (MND). Amyotrophic Lateral Sclerosis and Other Motor Neuron Disorders. 2002;3(3):123-9.
23. Matuz T, Birbaumer N, Hautzinger M, Kübler A. Psychosocial adjustment to ALS: a longitudinal study. Frontiers in psychology. 2015;6:1197.
24. King SJ, Duke MM, O'Connor BA. Living with amyotrophic lateral sclerosis/motor neurone disease (ALS/MND): decision-making about 'ongoing change and adaptation'. Journal of clinical nursing. 2009;18(5):745-54.
25. Locock L, Mazanderani F, Powell J. Metaphoric language and the articulation of emotions by people affected by motor neurone disease. Chronic illness. 2012;8(3):201-13.
26. Whitehead B, O'Brien MR, Jack BA, Mitchell D. Experiences of dying, death and bereavement in motor neurone disease: a qualitative study. Palliative Medicine. 2012;26(4):368-78.
27. Gould RL, Coulson MC, Brown RG, Goldstein LH, Al-Chalabi A, Howard RJ. Psychotherapy and pharmacotherapy interventions to reduce distress or improve well-being in people with amyotrophic lateral sclerosis: A systematic review. Amyotrophic Lateral Sclerosis and Frontotemporal Degeneration. 2015;16(5-6):293-302.
28. Tong A, Sainsbury P, Craig J. Consolidated criteria for reporting qualitative research (COREQ): a 32-item checklist for interviews and focus groups. International journal for quality in health care. 2007;19(6):349-57.
29. Braun V, Clarke V. Using thematic analysis in psychology. Qualitative research in psychology. 2006;3(2):77-101.
30. Braun V, Clarke V. Reflecting on reflexive thematic analysis. Qualitative Research in Sport, Exercise and Health. 2019;11(4):589-97.
31. Jenkinson C, Fitzpatrick R. Reduced item set for the amyotrophic lateral sclerosis assessment questionnaire: development and validation of the ALSAQ-5. Journal of Neurology, Neurosurgery & Psychiatry. 2001;70(1):70-3.
32. Foley G, Timonen V, Hardiman O. Exerting control and adapting to loss in amyotrophic lateral sclerosis. Social Science & Medicine. 2014;101:113-9.
33. Hugel H, Grundy N, Rigby S, Young CA. How does current care practice influence the experience of a new diagnosis of motor neuron disease? A qualitative study of current guidelines-based practice. Amyotrophic lateral sclerosis. 2006;7(3):161-6.
34. Oyebode JR, Smith H-J, Morrison K. The personal experience of partners of individuals with motor neuron disease. Amyotrophic Lateral Sclerosis and Frontotemporal Degeneration. 2013;14(1):39-43.
35. Galvin M, Corr B, Madden C, Mays I, McQuillan R, Timonen V, et al. Caregiving in ALS–a mixed methods approach to the study of burden. BMC palliative care. 2016;15(1):81.
36. Aoun SM, Bentley B, Funk L, Toye C, Grande G, Stajduhar KJ. A 10-year literature review of family caregiving for motor neurone disease: moving from caregiver burden studies to palliative care interventions. Palliative medicine. 2013;27(5):437-46.
37. Larsson BJ, Nordin K, Nygren I. Coping with amyotrophic lateral sclerosis; from diagnosis and during disease progression. Journal of the neurological sciences. 2016;361:235-42.
38. Jakobsson Larsson B, Nordin K, Askmark H, Nygren I. Coping strategies among patients with newly diagnosed amyotrophic lateral sclerosis. Journal of clinical nursing. 2014;23(21-22):3148-55.

For peer review only - http://bmjopen.bmj.com/site/about/guidelines.xhtml

39. Fanos JH, Gelinas DF, Foster RS, Postone N, Miller RG. Hope in palliative care: from narcissism to self-transcendence in amyotrophic lateral sclerosis. Journal of Palliative Medicine. 2008;11(3):470-5.
40. Foley G, Timonen V, Hardiman O. Understanding psycho-social processes underpinning engagement with services in motor neurone disease: a qualitative study. Palliative medicine. 2014;28(4):318-25.
41. Holkham L, Soundy A. The experience of informal caregivers of patients with motor neurone disease: a thematic synthesis. Palliative & supportive care. 2018;16(4):487-96.
42. Folkman S, Greer S. Promoting psychological well-being in the face of serious illness: when theory, research and practice inform each other. Psycho-Oncology: Journal of the Psychological, Social and Behavioral Dimensions of Cancer. 2000;9(1):11-9.
43. Pavey A, Warren N, Allen-Collinson J. "It gives me my freedom": Technology and responding to bodily limitations in motor neuron disease. Medical anthropology. 2015;34(5):442-55.
44. Weeks KR, Gould RL, Mcdermott C, Lynch J, Goldstein LH, Graham CD, et al. Needs and preferences for psychological interventions of people with motor neuron disease. Amyotrophic lateral sclerosis and frontotemporal degeneration. 2019;20(7-8):521-31.

For peer review only

Figure 1. Triggers of emotional distress

Figure 2. Strategies used to improve emotional wellbeing

For peer review only

For peer review only - http://bmjopen.bmj.com/site/about/guidelines.xhtml

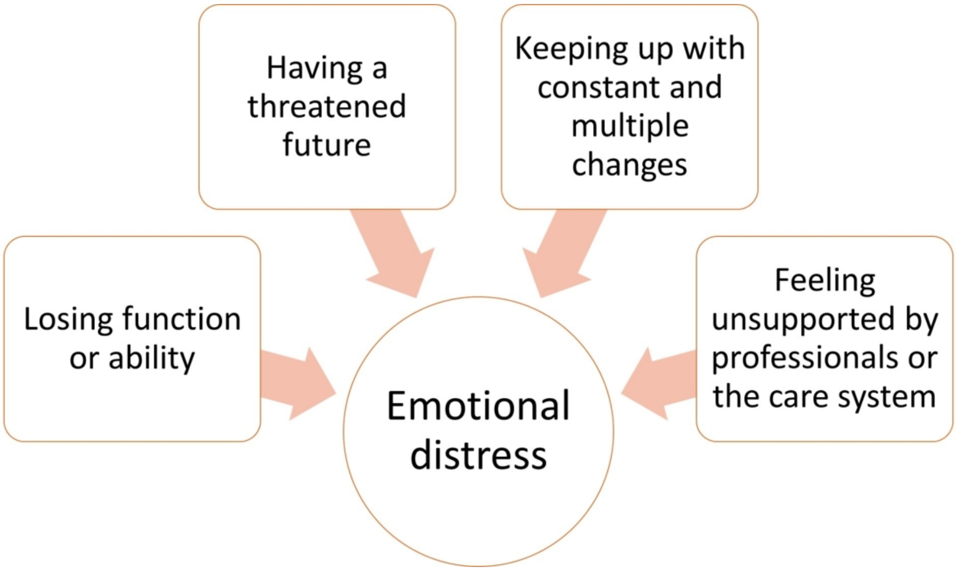

Figure 1. Triggers of emotional distress

167x94mm (300 x 300 DPI)

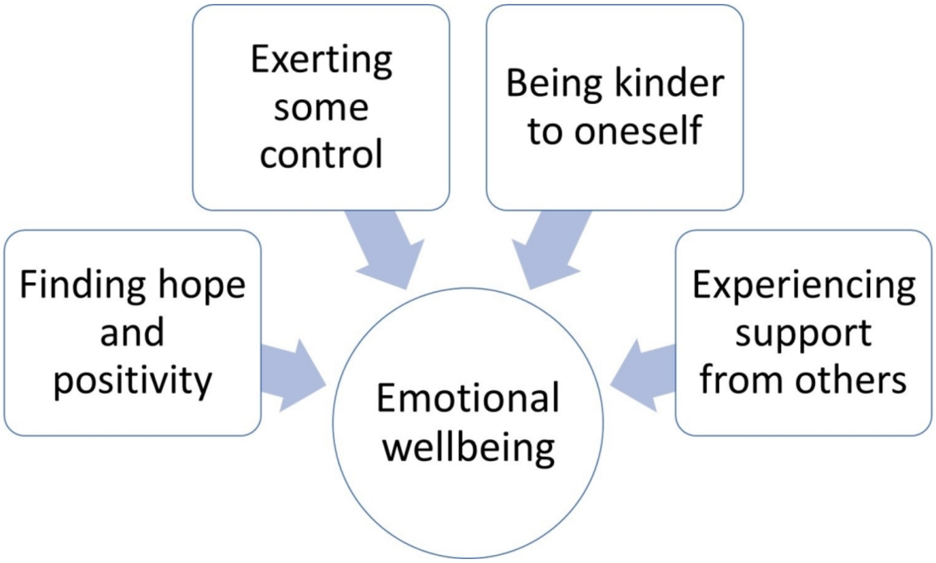

Figure 2. Strategies used to improve emotional wellbeing

427x236mm (96 x 96 DPI)

SUPPLEMENTARY FILE 1

1. Interview topic guide

(1) Can you tell me about your experience with MND?

- wherever the participant would like to start, could be about diagnosis or their experience now.
- Have your thoughts and feelings changed since you were diagnosed or are they the same? (If different, in what way have they changed?)

(2) What's it like to live with MND? Can you tell me a bit more about that?

- What are your main concerns/worries/emotions you face on a day to day basis?
- What are your thoughts and feelings about living with MND?

(3) Can you tell me about how you manage MND on a daily basis? If you find yourself having emotional concerns or worries, do you have any ways of managing them?

(4) Can you tell me about any support you feel you have? Or people/ things/ processes that have helped you?

(5) (In addition to this support) Do you have any thoughts about what else might support you to cope with MND?

For peer review only

- particularly additional support with any emotional concerns/worries that participants have previously mentioned.

For peer review only

SUPPLEMENTARY FILE 2

2. Consolidated criteria for reporting qualitative studies (COREQ):

Tong A, Sainsbury P, Craig J. Consolidated criteria for reporting qualitative research (COREQ): a 32-item checklist for interviews and focus groups. *International Journal for Quality in Health Care*. 2007. Volume 19, Number 6: pp. 349 – 357

No. Item	Guide questions/description	Reported on Page #
**Domain 1: Research team and reflexivity**		
Personal Characteristics		
1. Interviewer/facilitator	Which author/s conducted the interview or focus group?	CP – Methods section
2. Credentials	What were the researcher's credentials? E.g. PhD, MD	PhD student – Methods section
3. Occupation	What was their occupation at the time of the study?	PhD student – Methods section
4. Gender	Was the researcher male or female?	Female
5. Experience and training	What experience or training did the researcher have?	Methods section
Relationship with participants		
6. Relationship established	Was a relationship established prior to study commencement?	No
7. Participant knowledge of the interviewer	What did the participants know about the researcher? e.g. personal goals, reasons for doing the research	Participants knew that the researcher was a PhD student at the University of Southampton, doing her PhD on emotional distress and wellbeing in Motor Neurone Disease (MND). This information was described on the participant information sheets and

For peer review only

		study adverts.
8. Interviewer characteristics	What characteristics were reported about the interviewer/facilitator? e.g. Bias, assumptions, reasons and interests in the research topic	The interviewer did not have much prior knowledge of MND, but had previously worked with and interviewed people with Parkinson's disease and people receiving palliative care.
**Domain 2: study design**		
Theoretical framework		
9. Methodological orientation and Theory	What methodological orientation was stated to underpin the study? e.g. grounded theory, discourse analysis, ethnography, phenomenology, content analysis	The study used methods (in-depth semi-structured interviews and reflexive thematic analysis) in line with an interpretivist approach.
Participant selection		
10. Sampling	How were participants selected? e.g. purposive, convenience, consecutive, snowball	Purposive sampling – Methods section
11. Method of approach	How were participants approached? e.g. face-to-face, telephone, mail, email	Methods section
12. Sample size	How many participants were in the study?	Results section
13. Non-participation	How many people refused to participate or dropped out? Reasons?	Participants contacted the research if they wanted to take part in an interview. No one dropped out.
Setting		
14. Setting of data collection	Where was the data collected? e.g. home, clinic, workplace	Methods section
15. Presence of non-participants	Was anyone else present besides the participants and researchers?	No
16. Description of sample	What are the important characteristics of the sample? e.g. demographic data, date	Results section
Data collection		
17. Interview guide	Were questions, prompts, guides	Methods section and

For peer review only

	provided by the authors? Was it pilot tested?	supplementary file 1
18. Repeat interviews	Were repeat interviews carried out? If yes, how many?	N/A
19. Audio/visual recording	Did the research use audio or visual recording to collect the data?	Methods section
20. Field notes	Were field notes made during and/or after the interview or focus group?	Field notes were made after each interview.
21. Duration	What was the duration of the interviews or focus group?	Average duration reported – Results section
22. Data saturation	Was data saturation discussed?	No. We were not aiming for theoretical data saturation, but rather to look at patterns of experience across participants with diverse characteristics.
23. Transcripts returned	Were transcripts returned to participants for comment and/or correction?	No
**Domain 3: analysis and findings**		
Data analysis		
24. Number of data coders	How many data coders coded the data?	One – Methods section
25. Description of the coding tree	Did authors provide a description of the coding tree?	N/A
26. Derivation of themes	Were themes identified in advance or derived from the data?	Inductive thematic analysis – Methods section
27. Software	What software, if applicable, was used to manage the data?	NVivo
28. Participant checking	Did participants provide feedback on the findings?	Not participant checking, but a summary of findings was sent to all participants.
Reporting		
29. Quotations presented	Were participant quotations presented to illustrate the themes/findings? Was each quotation identified? e.g.	Yes, Results section

For peer review only

	participant number	
30. Data and findings consistent	Was there consistency between the data presented and the findings?	Yes, illustrative quotations have been provided for each theme and sub-theme – Results section
31. Clarity of major themes	Were major themes clearly presented in the findings?	Results section and presented in Figures 1 & 2
32. Clarity of minor themes	Is there a description of diverse cases or discussion of minor themes?	Description of diverse cases embedded within the explanation of themes – Results section

For peer review only

BMJ Open

Emotional distress and wellbeing among people with Motor Neurone Disease (MND) and their family caregivers: a qualitative interview study

Journal:	*BMJ Open*
Manuscript ID	bmjopen-2020-044724.R2
Article Type:	Original research
Date Submitted by the Author:	22-Jun-2021
Complete List of Authors:	Pinto, Cathryn; University of Southampton, Psychology<br>Geraghty, Adam; University of Southampton, Primary Care and Population Sciences<br>Yardley, Lucy; University of Southampton, Psychology; University of Bristol, School of Health Sciences<br>Dennison, Laura; University of Southampton
**Primary Subject Heading**:	Neurology
Secondary Subject Heading:	Qualitative research, Mental health
Keywords:	Motor neurone disease < NEUROLOGY, Depression & mood disorders < PSYCHIATRY, Anxiety disorders < PSYCHIATRY, QUALITATIVE RESEARCH

SCHOLARONE™
Manuscripts

I, the Submitting Author has the right to grant and does grant on behalf of all authors of the Work (as defined in the below author licence), an exclusive licence and/or a non-exclusive licence for contributions from authors who are: i) UK Crown employees; ii) where BMJ has agreed a CC-BY licence shall apply, and/or iii) in accordance with the terms applicable for US Federal Government officers or employees acting as part of their official duties; on a worldwide, perpetual, irrevocable, royalty-free basis to BMJ Publishing Group Ltd ("BMJ") its licensees and where the relevant Journal is co-owned by BMJ to the co-owners of the Journal, to publish the Work in this journal and any other BMJ products and to exploit all rights, as set out in our licence.

The Submitting Author accepts and understands that any supply made under these terms is made by BMJ to the Submitting Author unless you are acting as an employee on behalf of your employer or a postgraduate student of an affiliated institution which is paying any applicable article publishing charge ("APC") for Open Access articles. Where the Submitting Author wishes to make the Work available on an Open Access basis (and intends to pay the relevant APC), the terms of reuse of such Open Access shall be governed by a Creative Commons licence – details of these licences and which Creative Commons licence will apply to this Work are set out in our licence referred to above.

Other than as permitted in any relevant BMJ Author's Self Archiving Policies, I confirm this Work has not been accepted for publication elsewhere, is not being considered for publication elsewhere and does not duplicate material already published. I confirm all authors consent to publication of this Work and authorise the granting of this licence.

For peer review only

Emotional distress and wellbeing among people with Motor Neurone Disease (MND) and their family caregivers: a qualitative interview study

Cathryn Pinto,[a]* Adam W A Geraghty[b], Lucy Yardley[a,c] and Laura Dennison[a]

[a]*Department of psychology, University of Southampton, Southampton, UK;*

[b]*Primary care, population sciences and medical education, University of Southampton, Southampton, UK;*

[c]*School of psychological science, University of Bristol, Bristol, UK*

*Corresponding author: Cathryn Pinto, University of Southampton, B44 Highfield campus, Southampton SO17 1BJ Email: C.L.Pinto@soton.ac.uk

Word count: 3998

For peer review only - http://bmjopen.bmj.com/site/about/guidelines.xhtml

Abstract

Objective: We aimed to get an in-depth understanding of the emotions experienced by people with Motor Neurone Disease (MND) and their caregivers and to explore what impacts emotional distress and wellbeing.

Design: Qualitative study using semi-structured interviews with people with MND and caregivers.

Setting: Participants were recruited from across the UK and took part in interviews conducted either face-to-face, by telephone or email to accommodate for varying levels of disability.

Participants: 25 people with MND and 10 caregivers took part. Participants were purposively sampled based on their MND diagnosis, symptoms and time since diagnosis.

Data analysis: Data were analysed using inductive reflexive thematic analysis.

Results: Eight broad themes were generated (20 subthemes). Participants described the emotional distress of losing physical function and having a threatened future because of poor prognosis. Keeping up with constant changes in symptoms and feeling unsupported by the healthcare system added to emotional distress. Finding hope and positivity, exerting some control, being kinder to oneself, and experiencing support from others were helpful strategies for emotional wellbeing.

Conclusion: The study provides a broad understanding of what impacts emotional distress and wellbeing and discusses implications for psychological interventions for people with MND and caregivers. Any communication and support provided for people with MND and their caregivers, needs to pay attention to concepts of hope, control, and compassion.

For peer review only

Keywords: Motor neuron disease; emotional distress; wellbeing; depression; qualitative research

Article summary (Strengths and Limitations):

- This study provides an in-depth and inclusive account of what impacts emotional distress and wellbeing using interviews with people with MND and their family members.
- Purposive sampling and flexible recruitment strategies used to capture experiences of people with MND with a range of symptoms, including written interviews with who had difficulties with speech.
- Participants included people who had been recently diagnosed as well as those who had MND for years, and therefore captured experiences of emotional distress and wellbeing at different stages of having MND.
- Fewer people with mild cognitive impairment took part in the study.
- Study design did not allow us to see changes in emotional of distress and wellbeing over time, as symptoms change and people find new ways to adjust to changes.

For peer review only

INTRODUCTION

Motor neurone disease (MND) is a neurodegenerative disease, which results in declining physical function and has a very poor prognosis.(1) In terms of psychological impact, many people with MND (pwMND) experience depression.(2-4) Anxiety is prevalent around the time of diagnosis(5, 6) and during the final stages.(7) PwMND also experience feelings of hopelessness(8-10) and demoralization.(2)

Research has described the impact of the significant losses that come with MND on personal, social and occupational relationships.(10) Family members or caregivers of pwMND often struggle with the emotional impact and burden of the disease, and have high rates of psychological morbidity.(11-13) Studies with caregivers have described the strain from caring tasks, from having to make changes to their own lives and not having time for themselves.(14-16) Distress in MND has largely been described as anxiety and depression,(8, 12, 13, 17) or caregiver burden and strain.(18, 19) Wellbeing has largely been described as reduced anxiety and depression or improvements in quality of life.(20-22) In this paper, we use the terms 'emotional distress' to refer to the broad range of negative or difficult thoughts and emotions, and 'emotional wellbeing' for the broad range of positive or helpful thoughts and emotions experienced by pwMND and caregivers.

Studies have looked at demographic and clinical factors that might explain emotional distress or protect against it.(22) Results from these studies show that psychosocial factors like coping strategies and social support, are more strongly related to wellbeing and quality of life than demographic and clinical factors.(18, 22, 23) Emotions are also affected by low self-esteem,(4, 18) end of life concerns,(8) faith/existential concerns,(24, 25) sense of loss,(25, 26) and changes in identity, roles and relationships.(18, 25, 27) Psychosocial factors are clearly important for distress and wellbeing in MND, and we need a better understanding of

For peer review only - http://bmjopen.bmj.com/site/about/guidelines.xhtml

what influences emotional distress and the use of different coping strategies.(20, 28)

Qualitative research can be useful for understanding emotional distress and wellbeing because it allows participants to express their own understandings and experiences without being limited to concepts pre-determined by the researcher. Qualitative studies have largely explored the experience and impact of living with MND(10, 14, 25, 29), which have added to understanding of distress in MND. Few studies have focused specifically on the emotions experienced. One qualitative study examined the use of metaphors to express emotions,(30) and a small number have focused on emotions at specific time periods (e.g. during diagnosis or the final stages).(6, 26, 31) Emotional distress at other time points and things that influence distress and wellbeing need further exploration.

This study aims to build on our understanding of emotional distress and wellbeing in MND. More importantly, we aim to hear from people whose experiences are not well represented in research, including people with speech difficulties, cognitive impairment, and people at different stages of the disease. It is particularly timely to do this research because recent articles have highlighted that there are few effective psychological interventions to improve wellbeing,(32) and more research and interventions are needed.(12, 33) This study is part of a larger project to develop an intervention to improve emotional wellbeing among pwMND and caregivers.

In this current study, we aim to understand the emotional impact of living with MND and explore what impacts experiences of emotional distress and wellbeing among pwMND and their family caregivers.

For peer review only - http://bmjopen.bmj.com/site/about/guidelines.xhtml

METHODS

Design

Qualitative study using in-depth semi-structured interviews and reflexive thematic analysis, in line with an interpretivist approach. The paper is reported in accordance with the Consolidated criteria for reporting qualitative research (COREQ) (see Supplementary file 1).(34)

Participants

We aimed to recruit 20-30 pwMND and used purposive sampling to represent people with difficulties with movement, speech and cognition, and different lengths of time since diagnosis. Caregiver participants had fewer sampling criteria (age, gender), therefore we aimed to recruit 10-15 caregivers.

Eligibility: Participants were above 18 years of age, had an MND diagnosis, and had mental capacity to consider participation in the study (assessed by the researcher through correspondence about the study). We included participants who self-reported difficulties with cognition, but had mental capacity to give informed consent, as the views of pwMND who have cognitive impairment have been underrepresented in previous research. We included caregivers above 18 years of age, both current and recently bereaved (bereavement within 1 year from the time of the interview).

Data collection

Participants were recruited through a UK charity that supports pwMND and their families (Motor Neurone Disease Association). Study information was circulated via the charity's website, newsletters, social media outlets and local support groups. People willing to take part contacted CP, who screened for eligibility and provided further details about the interview.

For peer review only - http://bmjopen.bmj.com/site/about/guidelines.xhtml

Before each interview, participants gave written informed consent and filled a demographic/clinical details form. This process was completed either in-person, by post or email, based on the interview mode. CP, who has training and experience in conducting interviews for qualitative research, conducted all interviews. Interview mode was flexible (face-to-face, email or phone) to accommodate for various levels of disability. Face-to-face interviews were conducted in a place convenient to participants, usually at home or a hospice. Where two members of a couple had both consented to participate participants were interviewed separately where possible, but jointly where this was requested for reasons of comfort or to facilitate easier communication. The interview topic guide was developed iteratively by CP, LD, AG and patient and public involvement members. In line with an interpretivist approach, questions were broad and open-ended to allow participants to give rich, in-depth accounts of their emotional experiences in relation to MND, and follow-up questions were led by participants' responses. The final interview topic guide (Supplementary file 2) covered questions about people's experiences living with MND, with a focus on their thoughts and feelings and coping with emotional concerns.

Data analysis

Face-to-face and phone interviews were audio recorded and transcribed, and all interviews were anonymized. Field notes were completed after each interview, reflecting on participants' responses, and interview method and procedure. Data were analyzed using reflexive thematic analysis,(35, 36) as this was a flexible method that suited the research question, helped us identify common patterns across participants' experiences and allowed us to look for underlying meaning behind experiences of emotional distress and wellbeing. In line with an interpretivist approach, we used an inductive approach to data analysis, and included convergent and divergent cases in theme development. The analysis was mainly

For peer review only - http://bmjopen.bmj.com/site/about/guidelines.xhtml

conducted by CP, a PhD student with previous experience as a qualitative researcher. After familiarisation with the interview transcripts, Nvivo 12 software was used to code the data inductively, focusing on semantic and latent features of the data. The codes went through several iterations as new interviews were coded. During the coding process, notes were made about interesting features of the data and how different concepts related to each other. Following this, similar codes about emotional distress and emotional wellbeing were grouped together and candidate themes were generated. Themes were then reviewed to see if they represented experiences across the dataset. Theme names were revised and findings written up; this was also an iterative process whereby descriptions were clarified and overlap between themes identified. CP had regular meetings with qualitative research experts LD and AG who helped refine codes and themes. A lay summary of the findings was sent to all participants.

Ethics

We obtained ethics from the University of Southampton ethics committee (ERGOII-46996). Written informed consent was obtained for all participants. Participants were debriefed and steps were taken following each interview to ensure participants were not distressed.

Patient and Public Involvement (PPI)

Three PPI members (one person with MND and 2 former caregivers) contributed to this study. They were involved in the recruitment stage to help identify potential participants. They also helped pilot the interview topic guide, refine interview questions and trial the data collection procedure to ensure that it was not burdensome. Some PPI members looked over early drafts of the findings and offered insight about elements to highlight or discuss in the reporting of the results.

For peer review only - http://bmjopen.bmj.com/site/about/guidelines.xhtml

RESULTS

Participants were 25 pwMND and 10 family caregivers (see Table 1).

29 interviews were conducted in total; 6 joint interviews and 23 one-to-one interviews with either pwMND or caregiver . 14 interviews were conducted face-to-face, 8 via telephone, and 7 via email. Interviews lasted an average of 39.6 minutes.

Table 1: Demographic and clinical details of the sample

Characteristic	People with MND (n=25)	Caregivers (n=10)
**Age (range 39-80)**		
35-50 years	3	0
51-65 years	12	4
66-80 years	10	6
**Gender**		
Male	15	5
Female	10	5
**Relationship to person with MND**		
Spouse/partner		10
**Diagnosis**		
ALS limb	18	
ALS bulbar	1	
Primary Lateral Sclerosis	4	
Progressive Bulbar Palsy	1	

For peer review only - http://bmjopen.bmj.com/site/about/guidelines.xhtml

Progressive Muscular Atrophy	1
**Time since diagnosis (range 2 months-17 years)**	
Less than 1 year	8
1-3 years	6
3-5 years	7
More than 5 years	4
**Difficulties reported often/always *(ALSAQ-5 scale)**	
Difficult to stand up	16
Difficult to use arms and hands	16
Difficulty eating solid food	4
Speech is not easy to understand	9
Feeling hopeless about the future	3
**Self-reported concerns about cognitive ability**	2

*Amyotrophic Lateral Sclerosis Assessment Questionnaire (ALSAQ-5) is a patient self-report questionnaire used to briefly measure the impact of ALS/MND on patients.(37)

We developed 8 themes and 20 subthemes. The first four themes relate to triggers of emotional distress (Fig 1); the remaining four themes capture strategies to improve emotional wellbeing (Fig 2). All themes were present in some degree and form in both patient and caregiver interviews; where differences between these groups were apparent, they are described.

For peer review only - http://bmjopen.bmj.com/site/about/guidelines.xhtml

[Insert Fig 1 here]

Triggers of emotional distress

Participants described 4 main triggers of emotional distress and how they had an impact on emotions (themes are numbered, *subthemes in italics*).

(1) Losing function or ability

Most participants described how losing function or ability was distressing because of a *reduced sense of autonomy and control.* PwMND spoke about feelings of anger, frustration, sadness or grief at not being able to do the things they wanted to, at losing their independence and relying on others for help. Caregivers expressed similar emotions at seeing their family member lose capability and independence.

> "Hobbies that I had, I can't pursue any of them because I just don't have any capability enough in my arms now to do that. So, I think mentally I've seen a dip in the last six months into a much more kind of negative and angry stage really." (Person with MND, P13)

> "...and seeing him, and it saddened me, where you're sort of doing the job of hoisting and moving, the manual handling, and then you step back and you're seeing it from a distance, that's actually quite [pauses] hard as well, because he's got no control over his body and he's having to have people do that for him." (Caregiver, C05)

For peer review only - http://bmjopen.bmj.com/site/about/guidelines.xhtml

Losing function led to *changes in self-identity*, specifically those of becoming a 'patient' and 'carer'. These changes, especially losing one's voice which is strongly linked to identity, were experienced as distressing.

> "Losing mobility gradually was bad enough… but losing my voice is much more dreadful. It's taking away part of my personality. I can no longer express myself properly." (Person with MND, P21)

Losing function or ability sometimes led to *changes in relationships and interactions*. PwMND spoke about negative feelings of becoming a burden for their partner/spouse. Both patient and caregiver participants spoke about how having MND sometimes changed or limited interactions between family members.

> "It's emotionally difficult not being able to physically help my son or my parents and siblings and friends. Not being able to hold and play with my nephew." (Person with MND, P26)

Participants also had *practical concerns or worries about managing tasks* as a result of losing more ability and function.

> "My main worry at the moment really is moving him from there to there because his knees and lower legs are getting weaker and I've got that [points to hoist] to move him." (Caregiver, C03)

(2) Having a threatened future

For peer review only

The short prognosis that accompanies a diagnosis of MND led to feelings of anger, disappointment, sadness and feelings of being robbed/cheated of a future (*threat to life narrative and future plans*).

> "But I'm having to accept I might have a lonely old age. Disappointment, more than anything else.' (Caregiver, C27)

Participants also described emotional distress when *anticipating future symptoms or disease progression*. Many things triggered worry about the future including researching MND online, meeting other pwMND, discussions with healthcare professionals and going through symptom checklists. Some participants also spoke about experiencing such worries about the future even if symptoms had not progressed or were relatively stable. This was true particularly just after diagnosis, where worries about the future led to feelings of depression, low mood and withdrawal from others.

> "Even though physically, say that first month afterwards, there was almost no change but my mind's thinking quite morose almost. You almost think of death at that point... It's not even anger at that stage, it's just hopelessness at that point because you're just thinking oh that is it isn't it? It's all over." (Person with MND, P10)

The *uncertainty of the disease progression* also caused emotional distress among both patients and caregivers, because of being unable to manage symptoms or plan for the future.

For peer review only - http://bmjopen.bmj.com/site/about/guidelines.xhtml

> "I think the biggest thing about this is that it's such an unknown. Because everybody is different and, who knows how much, how long this disease is going to affect you. And the uncertainty, for me, it's very hard to cope with.' (Caregiver, C15)

(3) Keeping up with constant and multiple changes

Participants discussed how the *timing and relentless nature of changes* in symptoms was difficult physically and emotionally. As time went on, for some it became easier to fall into a care routine. However, if deterioration happened quickly, both patient and caregivers expressed that it was difficult to cope and left them feeling tired, not in control and in need of respite.

> "If something's difficult, we find a way round it. And by the time we've found a way round it, things have moved on again, and it doesn't work. So that's very frustrating. And a bit… not depressing… soul destroying, because we're always playing catch up." (Person with MND, P24)

Sometimes physical symptoms necessitated *changes in many areas of life.* This included changes to their home, work, social and leisure activities. Both pwMND and caregivers spoke about the effort involved in either living with MND or looking after someone with MND. This sometimes affected thoughts and mood negatively including feelings of frustration, anxiety, tiredness or strain/burden. For example, C03, a caregiver, recalled being upset and needing someone to talk to due to exhaustion from physically moving her spouse; "I used to get so tired that sometimes when he had gone to bed in the evening I did just feel like standing there crying."

For peer review only - http://bmjopen.bmj.com/site/about/guidelines.xhtml

(4) Feeling unsupported by professionals or the care system

Participants reported feeling distressed when healthcare professionals or the care system did not support their needs. *Infrequent or inefficient care provision* left people feeling abandoned and left on their own to deal with MND.

> "I felt completely unsupported by the neurologist or the health service, we were really just left on our own, completely in the dark as to what was gonna happen... So, it was a very, very distressing time." (Person with MND, P14)

Poor coordination between different professionals/organizations, difficulty accessing equipment and financial benefits also triggered feelings of frustration, injustice and being 'let down' by the system.

Poor or insensitive communication from healthcare professionals added to emotional distress. In particular, participants described upset as a consequence of a lack of empathy from professionals, or insensitive or unsupportive presentation of care.

> "Our life was shattered in 2 minutes. And I felt that was cruel, very very cruel [pause]. There should be a way of discussing it with you and saying "well look, you have such and such a condition, but hey, this is what we can do." Not just you've got this and throw you out, and so we had to find our own way round this problem." (Caregiver, C01)

For peer review only - http://bmjopen.bmj.com/site/about/guidelines.xhtml

[Insert Fig. 2 here]

Strategies used to improve emotional wellbeing

Participants described strategies they used to improve emotional wellbeing (themes are numbered; *subthemes in italics*).

(1) Finding hope and positivity

Despite the many losses, participants actively found ways to introduce hope and positivity. For many people, acceptance of MND was a gradual, learned process and even once there was acceptance, an attitude of not giving up on life was important for coping (*acceptance and not giving up)*.

> "I always wanna think I've got options, got somewhere to go because think at the point where you think well I've got nowhere left to go with this, that's when you may sort of deteriorate and let it get the best of you. But I think if you if you say right well I'm going to, you know, there's this trial, this might happen, I'll go and see a physio… and just see if that helps me." (Person with MND, P18)

For some, not giving up included hoping for a cure or trying alternative therapies. For others, not giving up included finding solutions to make life easier, and not letting MND define them as they continued normal and valued activities. This strategy was endorsed mainly by pwMND.

> "not giving up, not allowing it to put you away in a corner somewhere, waiting for the inevitable… not allowing that to define what you do with whatever time you have left." (Person with MND, P06)

For peer review only - http://bmjopen.bmj.com/site/about/guidelines.xhtml

Despite severe losses of function, many participants continued *doing activities that they enjoyed and that were meaningful*. Planning ahead and having things to look forward to in the immediate future added to feelings of hope.

> "Key thing is to have lots of little goals, lots of little hopes of things to do, normalities, weekends away, holidays but not in the far future. Everything's in a couple of weeks, 2-3 weeks, something to look forward to… it keeps the mind occupied to know that there is something coming up." (Person with MND, P10)

Most participants tried to *focus on the positive aspects of life*. This strategy became difficult in the context of increasing loss, but participants still stressed that it was important.

> "I'm very much sort of an optimist. You can't…you know, because it's horrible we know that. But we just make [name of pwMND] as comfortable as possible. You've got to be positive, otherwise you just sink and go under." (Caregiver, C07)

To stay positive and deal with negative emotions, participants tried to reframe difficult situations by focusing on what they still can do, using humour, feeling grateful for symptoms they did not have or feeling lucky because of their circumstances or because of the support they received.

For peer review only - http://bmjopen.bmj.com/site/about/guidelines.xhtml

(2) Exerting some control

When MND took some control away, participants found other ways to exert control over their lives. Although a sense of control was important for most participants, they exerted this control in very different ways. One way was by *focusing on the present moment* and making the most of what they could currently do. This strategy was adopted mainly by pwMND; it stopped them from worrying about the future or feeling sad for what they could no longer do, and instead had a more positive impact on emotions.

> "I realised there's absolutely no point in dwelling on the past because there's absolutely nothing I can do to get that back. So, it's waste of energy and there's no point in worrying about the future because there's absolutely nothing I can do about it. So, what I kind of taught myself to do is try to live in here and now because I do have some control over that." (Person with MND, P04)

Some participants described how it was helpful to exert *control over decisions about their care,* organizing or planning care and taking a more active problem-solving approach. This gave people a sense of empowerment when they would otherwise be struggling to cope, and also helped people hold onto their independence or identity despite the loss.

> "I hate the fact that I can't talk. Although I can talk, but it's a bit drained and doesn't sound like me. But I've done voice banking and I think you've got to be a step ahead." (Person with MND, P19)

For peer review only - http://bmjopen.bmj.com/site/about/guidelines.xhtml

> ‘they’re [professionals] pretty good at picking up what might be the next thing that’s necessary or how tired I’m looking and dropping in a suggestion here and there about what you might change, what you might need next… in terms of future proofing, so you’re on top of a problem and not floundering about solving it.’ (Caregiver, C05)

However, some people exerted control by avoiding interactions or information that might remind them of future symptoms or deterioration and associated negative emotions.

> “Everyone’s different, aren’t they? And their MND is different. Some people like to know the ins and out, but I don’t know, I like to bury my head in the sand. Because I know that I would sit and worry about it all the time. And I don’t want to.” (Person with MND, P24)

(3) Being kinder to oneself

This strategy was mentioned by both pwMND and caregivers but for different kinds of tasks. Living with MND was often described as overwhelming and participants expressed the need for *taking a break* from MND. Caregivers spoke about the need to take a physical break from MND care; while pwMND spoke about taking a break from MND by doing regular activities and not thinking about MND.

> “I’ll have days where I deliberately avoid looking at anything and trying just to have days or a couple of days where I don’t think about MND.” (Person with MND, P18)

For peer review only - http://bmjopen.bmj.com/site/about/guidelines.xhtml

Some participants also expressed the need to give themselves *time and space to adjust to changes* in symptoms. This was done by allowing themselves space to vent if they felt overwhelmed, pacing their activities, lowering expectations, modifying how tasks were done, or asking for help.

> "Keep doing the things you enjoy, just lower your expectations and standards so you achieve and don't feel disappointed." (Person with MND, P21)

(4) Experiencing support from others

Emotional and practical support from healthcare professionals, friends and family helped pwMND and caregivers cope with any emotional distress. Emotional support included being empathic, listening, being encouraging and positive. Participants also valued support from other people with MND because they felt truly understood and less alone.

> "I had a confirmation diagnosis at [hospital] and then I had a phone call from the [hospice] asking if I wanted to attend a kind of MND first contact group... and it's been a fantastic thing and I still go now. And that was really good, really supportive." (Person with MND, P19)

There were some differing views about the timing of offering support, especially just after diagnosis. Some participants wanted access to information and support almost immediately, however others needed some time to adjust before they accessed support.

Supportive communication from healthcare professionals and reliability of care helped patients and families feel reassured and confident about their care, which led to positive

For peer review only - http://bmjopen.bmj.com/site/about/guidelines.xhtml

experiences and emotions. This included providing information based on the patient and families' readiness, communicating information in a sensitive and empathic manner, focusing on what can be done in terms of care and timely provision of care and equipment.

> "I would also mention my occupational therapist who has been brilliant at assessing my needs and getting in equipment quickly, usually just before they were needed. This has given me more confidence in the care and support I am given." (Person with MND, P22)

For peer review only - http://bmjopen.bmj.com/site/about/guidelines.xhtml

DISCUSSION

This study advances our understanding of what influences emotional distress and wellbeing among people with MND and their caregivers. Our findings come from a diverse sample, thereby highlighting key triggers of distress and coping strategies used by people with different abilities, symptoms and at different stages of having MND. PwMND and caregivers use coping strategies differently but still rely on similar concepts (hope, positivity, control, self-kindness, social support) to improve emotional wellbeing.

Some of our findings are in line with previous MND research, such as the distress caused by loss of ability and threats to the future.(10, 26, 27, 38) Previous research has also identified distress caused through not receiving appropriate professional support.(6, 7, 10, 31, 39, 40) We highlight the emotional distress triggered by multiple and constant changes brought about by disease progression and constantly ‘playing catch up.’ This is a new finding in terms of emotional distress for pwMND, but has been described in the literature around caregiver experience.(17, 41, 42) This is an important finding for intervention development, and we need to ensure that interventions are perceived as manageable, not burdensome.
In terms of emotional wellbeing, hope and control are particularly important for MND.(10, 38, 40, 43, 44) Soundy and Condon (2015) have developed a model to show how concepts of hope and control can affect mental wellbeing in MND.(10) Hope and control can be difficult concepts to apply in MND where hope and control are constantly being threatened. Our findings highlight these complexities, and provide examples of how pwMND and caregivers use hope and control to maintain and improve wellbeing, despite this threat. For example, we found that many people use meaning-based coping strategies by either re-appraising difficult circumstances in a positive way or doing activities that were important and meaningful. However, for some accepting their circumstances was difficult and hope meant adopting an

For peer review only - http://bmjopen.bmj.com/site/about/guidelines.xhtml

attitude of not giving up and not letting MND define them. Psychological interventions such as acceptance and commitment therapy can be useful and can foster meaning-based coping strategies.(45-47) However, there might be certain challenges in how interventions are presented to people who may find acceptance difficult with MND. Our findings also show how control is exerted differently in the face of loss, by focusing more on the present moment, or by making decisions about receiving information and care. This has implications for support services, especially in providing options for care, equipment and communication aids.(48) The importance of focusing on the present demonstrates the value of mindfulness-based approaches for psychological interventions. The differences in how people use control highlights the importance of having flexible support that is tailored to the individual's needs and coping preferences.(47) Another novel finding in relation to emotional wellbeing and MND is the importance of self-kindness and self-care. Self-compassion has been associated with adaptive coping strategies and wellbeing other chronic illnesses (49-51) and similar approaches may be useful for pwMND and their caregivers.

Strengths and Limitations

We aimed to recruit a diverse range of participants and succeeded in sampling people with different symptoms of varying severities, particularly people with difficulties speaking. We did not manage to recruit many people with mild cognitive impairment and could have benefitted from a more targeted sampling approach. Although we captured the experiences of newly diagnosed people and people who had MND for several years, we could not ascertain if we captured the experiences of people who were at the end stages. Conducting email interviews ensured we included the experiences of people with speech difficulties; however there were some methodological difficulties in using prompts and asking follow up questions. Despite these limitations, including the experiences of under-represented groups gives us

For peer review only

confidence in the application of these findings for people with different symptoms and at different stages of MND.

Conclusion

We provide a broad understanding of what impacts emotional distress and wellbeing among pwMND and their caregivers. Findings have important implications for psychological interventions, services and professionals that support pwMND and their families. Any communication and support provided for pwMND and their caregivers, needs to pay attention to concepts of hope, control, and compassion, and how individuals may use these concepts differently to cope with the emotional impact of MND.

For peer review only

For peer review only - http://bmjopen.bmj.com/site/about/guidelines.xhtml

Funding statement:

This work was supported by the Motor Neurone Disease Association, UK under Grant [Yardley/May17/891-792].

Competing interest statement:

The authors report no conflict of interest.

Data availability statement:

Anonymised interview transcripts can be made available upon reasonable request from the corresponding author Cathryn Pinto (C.L.Pinto@soton.ac.uk).

Author contributions:

Cathryn Pinto: Main contributor to the design of the study, ethics application, recruitment and data collection, data analysis, reporting and publication of findings.

Dr. Adam Geraghty: Involved in obtaining funding for the study, contributed to the study design, supervised Cathryn during recruitment, data collection and data analysis, and contributed to the reporting and publication of findings.

Prof. Lucy Yardley: Involved in obtaining funding for the study, contributed to the study design and the reporting and publication of findings.

Dr. Laura Dennison: Involved in obtaining funding for the study, contributed to the study design, supervised Cathryn during recruitment, data collection and data analysis, and contributed to the reporting and publication of findings.

For peer review only - http://bmjopen.bmj.com/site/about/guidelines.xhtml

References

1. Moore C, McDermott CJ, Shaw PJ. Clinical aspects of motor neurone disease. Medicine. 2008;36(12):640-5.
2. Clarke DM, McLeod JE, Smith GC, Trauer T, Kissane DW. A comparison of psychosocial and physical functioning in patients with motor neurone disease and metastatic cancer. Journal of palliative care. 2005;21(3):173-9.
3. Goldstein LH, Adamson M, Jeffrey L, Down K, Barby T, Wilson C, et al. The psychological impact of MND on patients and carers. Journal of the Neurological Sciences. 1998;160:S114-S21.
4. Hogg K, Goldstein LH, Leigh P. The psychological impact of motor neurone disease. Psychological medicine. 1994;24(3):625-32.
5. O'Brien MR, Whitehead B, Jack BA, Mitchell JD. From symptom onset to a diagnosis of amyotrophic lateral sclerosis/motor neuron disease (ALS/MND): experiences of people with ALS/MND and family carers–a qualitative study. Amyotrophic lateral sclerosis. 2011;12(2):97-104.
6. Pavey A, Allen-Collinson J, Pavey T. The lived experience of diagnosis delivery in motor neurone disease: a sociological-phenomenological study. Sociological research online. 2013;18(2):36-47.
7. O'Brien MR, Whitehead B, Jack BA, Mitchell JD. The need for support services for family carers of people with motor neurone disease (MND): views of current and former family caregivers a qualitative study. Disability and rehabilitation. 2012;34(3):247-56.
8. Averill AJ, Kasarskis EJ, Segerstrom SC. Psychological health in patients with amyotrophic lateral sclerosis. Amyotrophic Lateral Sclerosis. 2007;8(4):243-54.
9. McLeod JE, Clarke DM. A review of psychosocial aspects of motor neurone disease. Journal of the neurological sciences. 2007;258(1-2):4-10.
10. Soundy A, Condon N. Patients experiences of maintaining mental well-being and hope within motor neuron disease: a thematic synthesis. Frontiers in psychology. 2015;6:606.
11. Goldstein LH, Leigh PN. Motor neurone disease: a review of its emotional and cognitive consequences for patients and its impact on carers. British Journal of Health Psychology. 1999;4(3):193-208.
12. Pagnini F. Psychological wellbeing and quality of life in amyotrophic lateral sclerosis: a review. International Journal of Psychology. 2013;48(3):194-205.
13. Rabkin JG, Albert SM, Rowland LP, Mitsumoto H. How common is depression among ALS caregivers? A longitudinal study. Amyotrophic Lateral Sclerosis. 2009;10(5-6):448-55.
14. Cox DL. Perspectives of motor neurone disease. Clinical rehabilitation. 1992;6(4):333-9.
15. Brown JB. User, carer and professional experiences of care in motor neurone disease. Primary Health Care Research & Development. 2003;4(3):207-17.
16. Olsson Ozanne AG, Strang S, Persson LI. Quality of life, anxiety and depression in ALS patients and their next of kin. Journal of clinical nursing. 2011;20(1-2):283-91.
17. Galvin M, Corr B, Madden C, Mays I, McQuillan R, Timonen V, et al. Caregiving in ALS–a mixed methods approach to the study of burden. BMC palliative care. 2016;15(1):81.
18. Goldstein L, Atkins L, Landau S, Brown R, Leigh P. Longitudinal predictors of psychological distress and self-esteem in people with ALS. Neurology. 2006;67(9):1652-8.
19. de Wit J, Bakker LA, van Groenestijn AC, van den Berg LH, Schröder CD, Visser-Meily JMA, et al. Caregiver burden in amyotrophic lateral sclerosis: A systematic review. Palliat Med. 2018;32(1):231-45.

For peer review only - http://bmjopen.bmj.com/site/about/guidelines.xhtml

20. Larsson BJ, Nordin K, Nygren I. Coping with amyotrophic lateral sclerosis; from diagnosis and during disease progression. Journal of the neurological sciences. 2016;361:235-42.
21. Matuz T, Birbaumer N, Hautzinger M, Kübler A. Coping with amyotrophic lateral sclerosis: an integrative view. Journal of Neurology, Neurosurgery & Psychiatry. 2010;81(8):893-8.
22. Matuz T, Birbaumer N, Hautzinger M, Kübler A. Psychosocial adjustment to ALS: a longitudinal study. Frontiers in psychology. 2015;6:1197.
23. Goldstein L, Atkins L, Leigh P. Correlates of quality of life in people with motor neuron disease (MND). Amyotrophic Lateral Sclerosis and Other Motor Neuron Disorders. 2002;3(3):123-9.
24. Bolmsjö I. Existential issues in palliative care: interviews of patients with amyotrophic lateral sclerosis. Journal of palliative medicine. 2001;4(4):499-505.
25. Foley G, O'Mahony P, Hardiman O. Perceptions of quality of life in people with ALS: effects of coping and health care. Amyotrophic lateral sclerosis. 2007;8(3):164-9.
26. Mistry K, Simpson J. Exploring the transitional process from receiving a diagnosis to living with motor neurone disease. Psychology & health. 2013;28(8):939-53.
27. Sakellariou D, Boniface G, Brown P. Experiences of living with motor neurone disease: a review of qualitative research. Disability and rehabilitation. 2013;35(21):1765-73.
28. Fisher P, Dodd R, Barrow E, Makin S, Cherry MG. Predictors of distress in amyotrophic lateral sclerosis: A systematic review. Cogent Psychology. 2019;6(1):1608031.
29. King SJ, Duke MM, O’Connor BA. Living with amyotrophic lateral sclerosis/motor neurone disease (ALS/MND): decision-making about ‘ongoing change and adaptation’. Journal of clinical nursing. 2009;18(5):745-54.
30. Locock L, Mazanderani F, Powell J. Metaphoric language and the articulation of emotions by people affected by motor neurone disease. Chronic illness. 2012;8(3):201-13.
31. Whitehead B, O’Brien MR, Jack BA, Mitchell D. Experiences of dying, death and bereavement in motor neurone disease: a qualitative study. Palliative Medicine. 2012;26(4):368-78.
32. Gould RL, Coulson MC, Brown RG, Goldstein LH, Al-Chalabi A, Howard RJ. Psychotherapy and pharmacotherapy interventions to reduce distress or improve well-being in people with amyotrophic lateral sclerosis: A systematic review. Amyotrophic Lateral Sclerosis and Frontotemporal Degeneration. 2015;16(5-6):293-302.
33. Zarotti N, Mayberry E, Ovaska-Stafford N, Eccles F, Simpson J. Psychological interventions for people with motor neuron disease: a scoping review. Amyotroph Lateral Scler Frontotemporal Degener. 2021;22(1-2):1-11.
34. Tong A, Sainsbury P, Craig J. Consolidated criteria for reporting qualitative research (COREQ): a 32-item checklist for interviews and focus groups. International journal for quality in health care. 2007;19(6):349-57.
35. Braun V, Clarke V. Using thematic analysis in psychology. Qualitative research in psychology. 2006;3(2):77-101.
36. Braun V, Clarke V. Reflecting on reflexive thematic analysis. Qualitative Research in Sport, Exercise and Health. 2019;11(4):589-97.
37. Jenkinson C, Fitzpatrick R. Reduced item set for the amyotrophic lateral sclerosis assessment questionnaire: development and validation of the ALSAQ-5. Journal of Neurology, Neurosurgery & Psychiatry. 2001;70(1):70-3.
38. Foley G, Timonen V, Hardiman O. Exerting control and adapting to loss in amyotrophic lateral sclerosis. Social Science & Medicine. 2014;101:113-9.

For peer review only - http://bmjopen.bmj.com/site/about/guidelines.xhtml

39. Hugel H, Grundy N, Rigby S, Young CA. How does current care practice influence the experience of a new diagnosis of motor neuron disease? A qualitative study of current guidelines-based practice. Amyotrophic lateral sclerosis. 2006;7(3):161-6.
40. Holkham L, Soundy A. The experience of informal caregivers of patients with motor neurone disease: a thematic synthesis. Palliative & supportive care. 2018;16(4):487-96.
41. Oyebode JR, Smith H-J, Morrison K. The personal experience of partners of individuals with motor neuron disease. Amyotrophic Lateral Sclerosis and Frontotemporal Degeneration. 2013;14(1):39-43.
42. Aoun SM, Bentley B, Funk L, Toye C, Grande G, Stajduhar KJ. A 10-year literature review of family caregiving for motor neurone disease: moving from caregiver burden studies to palliative care interventions. Palliative medicine. 2013;27(5):437-46.
43. Fanos JH, Gelinas DF, Foster RS, Postone N, Miller RG. Hope in palliative care: from narcissism to self-transcendence in amyotrophic lateral sclerosis. Journal of Palliative Medicine. 2008;11(3):470-5.
44. Foley G, Timonen V, Hardiman O. Understanding psycho-social processes underpinning engagement with services in motor neurone disease: a qualitative study. Palliative medicine. 2014;28(4):318-25.
45. Graham CD, Simmons Z, Stuart SR, Rose MR. The potential of psychological interventions to improve quality of life and mood in muscle disorders. Muscle & Nerve. 2015;52(1):131-6.
46. Kangas M, McDonald S. Is it time to act? The potential of acceptance and commitment therapy for psychological problems following acquired brain injury. Neuropsychol. 2011;21(2):250-76.
47. Weeks KR, Gould RL, Mcdermott C, Lynch J, Goldstein LH, Graham CD, et al. Needs and preferences for psychological interventions of people with motor neuron disease. Amyotrophic lateral sclerosis and frontotemporal degeneration. 2019;20(7-8):521-31.
48. Pavey A, Warren N, Allen-Collinson J. “It gives me my freedom”: Technology and responding to bodily limitations in motor neuron disease. Medical anthropology. 2015;34(5):442-55.
49. Sirois FM, Molnar DS, Hirsch JK. Self-compassion, stress, and coping in the context of chronic illness. Self and Identity. 2015;14(3):334-47.
50. Wren AA, Somers TJ, Wright MA, Goetz MC, Leary MR, Fras AM, et al. Self-compassion in patients with persistent musculoskeletal pain: relationship of self-compassion to adjustment to persistent pain. Journal of pain and symptom management. 2012;43(4):759-70.
51. Pinto-Gouveia J, Duarte C, Matos M, Fráguas S. The protective role of self-compassion in relation to psychopathology symptoms and quality of life in chronic and in cancer patients. Clinical psychology & psychotherapy. 2014;21(4):311-23.

Figure 1. Triggers of emotional distress

Figure 2. Strategies used to improve emotional wellbeing

For peer review only - http://bmjopen.bmj.com/site/about/guidelines.xhtml

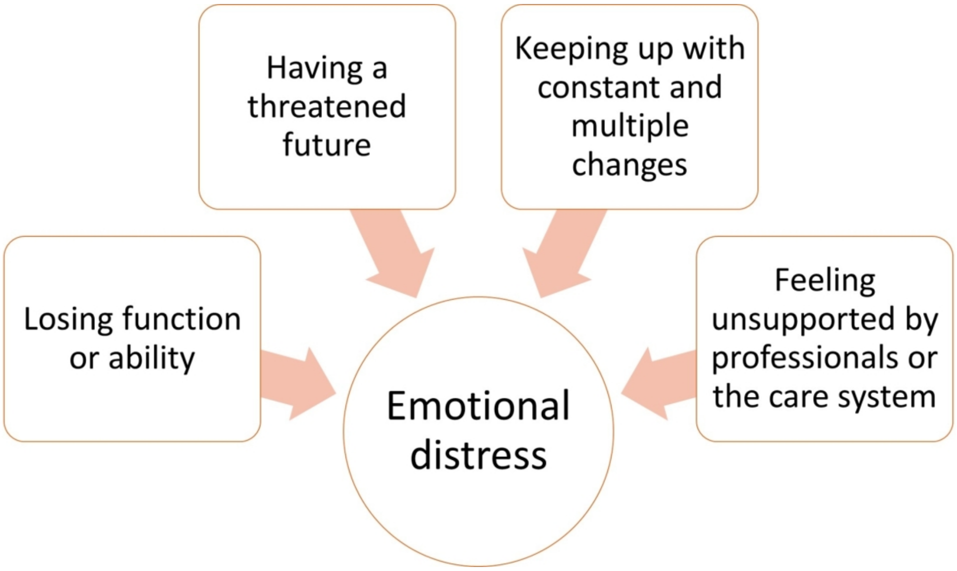

Figure 1. Triggers of emotional distress

167x94mm (300 x 300 DPI)

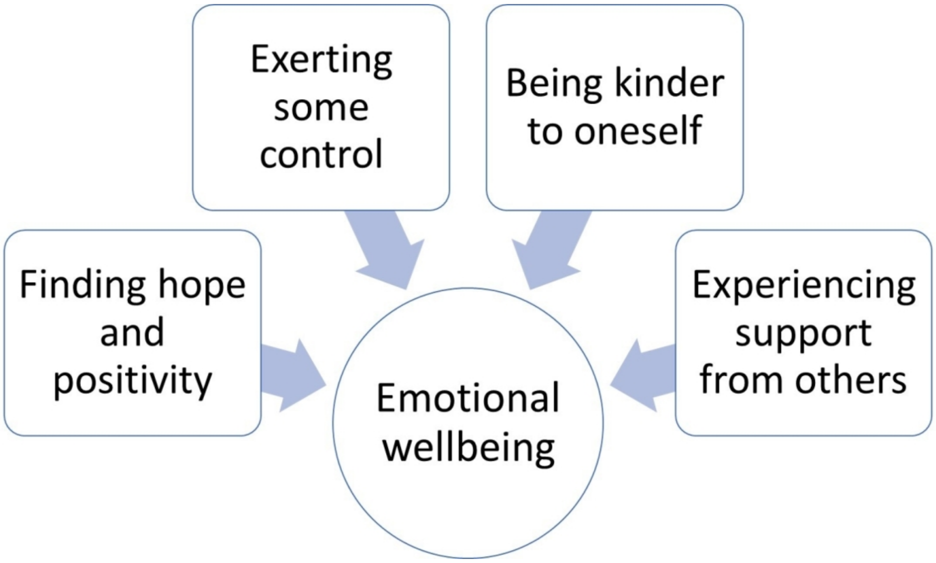

Figure 2. Strategies used to improve emotional wellbeing

427x236mm (96 x 96 DPI)

SUPPLEMENTARY FILE 1

2. Consolidated criteria for reporting qualitative studies (COREQ):

Tong A, Sainsbury P, Craig J. Consolidated criteria for reporting qualitative research (COREQ): a 32-item checklist for interviews and focus groups. *International Journal for Quality in Health Care*. 2007. Volume 19, Number 6: pp. 349 – 357

No. Item	Guide questions/description	Reported on Page #
**Domain 1: Research team and reflexivity**		
Personal Characteristics		
1. Interviewer/facilitator	Which author/s conducted the interview or focus group?	CP – Methods section, Pg 7
2. Credentials	What were the researcher's credentials? E.g. PhD, MD	PhD student – Methods section, Pg 8
3. Occupation	What was their occupation at the time of the study?	PhD student – Methods section, Pg 8
4. Gender	Was the researcher male or female?	Female
5. Experience and training	What experience or training did the researcher have?	Methods section – Pg 7-8
Relationship with participants		
6. Relationship established	Was a relationship established prior to study commencement?	No, Pg 6
7. Participant knowledge of the interviewer	What did the participants know about the researcher? e.g. personal goals, reasons for doing the research	Participants knew that the researcher was a PhD student at the University of Southampton, doing her PhD on emotional distress and wellbeing in Motor Neurone Disease (MND). This information was described on the participant information sheets and

For peer review only

		study adverts. Pg 6
8. Interviewer characteristics	What characteristics were reported about the interviewer/facilitator? e.g. Bias, assumptions, reasons and interests in the research topic	The interviewer did not have much prior knowledge of MND, but had previously worked with and interviewed people with Parkinson's disease and people receiving palliative care. Pg 8
**Domain 2: study design**		
Theoretical framework		
9. Methodological orientation and Theory	What methodological orientation was stated to underpin the study? e.g. grounded theory, discourse analysis, ethnography, phenomenology, content analysis	The study used methods (in-depth semi-structured interviews and reflexive thematic analysis) in line with an interpritivist paradigm. Pg 6
Participant selection		
10. Sampling	How were participants selected? e.g. purposive, convenience, consecutive, snowball	Purposive sampling – Methods section, Pg 6
11. Method of approach	How were participants approached? e.g. face-to-face, telephone, mail, email	Methods section – Pg 6-7
12. Sample size	How many participants were in the study?	Methods section – Pg 9
13. Non-participation	How many people refused to participate or dropped out? Reasons?	Participants contacted the researcher if they wanted to take part in an interview. No one dropped out. Pg 9
Setting		
14. Setting of data collection	Where was the data collected? e.g. home, clinic, workplace	Methods section – Pg 7
15. Presence of non-participants	Was anyone else present besides the participants and researchers?	No (not reported because not present)
16. Description of sample	What are the important characteristics of the sample? e.g. demographic data, date	Methods section – Pgs 9-10
Data collection		

For peer review only

17. Interview guide	Were questions, prompts, guides provided by the authors? Was it pilot tested?	Methods section, Pg 7 and supplementary file 2
18. Repeat interviews	Were repeat interviews carried out? If yes, how many?	N/A
19. Audio/visual recording	Did the research use audio or visual recording to collect the data?	Methods section – Pg 7
20. Field notes	Were field notes made during and/or after the interview or focus group?	Field notes were made after each interview. Pg 7
21. Duration	What was the duration of the interviews or focus group?	Average duration reported – Methods section, Pg 9
22. Data saturation	Was data saturation discussed?	No. We were not aiming for theoretical data saturation, but rather to look at patterns of experience across participants with diverse characteristics. (not reported because not applicable to this study)
23. Transcripts returned	Were transcripts returned to participants for comment and/or correction?	No (not reported because was not carried out)
**Domain 3: analysis and findings**		
Data analysis		
24. Number of data coders	How many data coders coded the data?	One – Methods section, Pgs 7-8
25. Description of the coding tree	Did authors provide a description of the coding tree?	N/A
26. Derivation of themes	Were themes identified in advance or derived from the data?	Inductive thematic analysis – Methods section, Pg 7
27. Software	What software, if applicable, was used to manage the data?	NVivo Pg 8
28. Participant checking	Did participants provide feedback on the findings?	Not participant checking, but lay summary of findings was sent to all participants Pg 8
Reporting		

For peer review only

29. Quotations presented	Were participant quotations presented to illustrate the themes/findings? Was each quotation identified? e.g. participant number	Yes, Results section Pgs 9-21
30. Data and findings consistent	Was there consistency between the data presented and the findings?	Yes, illustrative quotations have been provided for each theme and sub-theme – Results section Pgs 9-21
31. Clarity of major themes	Were major themes clearly presented in the findings?	Results section and presented in Figures 1 & 2, Pgs 9-21
32. Clarity of minor themes	Is there a description of diverse cases or discussion of minor themes?	Description of diverse cases embedded within the explanation of themes – Results section Pgs 9-21

For peer review only

SUPPLEMENTARY FILE 2

1. Interview topic guide

(1) Can you tell me about your experience with MND?

- wherever the participant would like to start, could be about diagnosis or their experience now.
- Have your thoughts and feelings changed since you were diagnosed or are they the same? (If different, in what way have they changed?)

(2) What's it like to live with MND? Can you tell me a bit more about that?

- What are your main concerns/worries/emotions you face on a day to day basis?
- What are your thoughts and feelings about living with MND?

(3) Can you tell me about how you manage MND on a daily basis? If you find yourself having emotional concerns or worries, do you have any ways of managing them?

(4) Can you tell me about any support you feel you have? Or people/ things/ processes that have helped you?

(5) (In addition to this support) Do you have any thoughts about what else might support you to cope with MND?

For peer review only

- particularly additional support with any emotional concerns/worries that participants have previously mentioned.

For peer review only
